# Active adults have thicker peripheral muscles and diaphragm: A cross-sectional study

**DOI:** 10.12688/f1000research.135379.1

**Published:** 2023-07-17

**Authors:** Aishwarya Shetty, Baskaran Chandrasekaran, Koustubh Kamath, Sneha Ravichandran, Rajagopal Kadavigere, Leena R David, Banumathe Karuppaya, Guruprasad Vijayasarathi, Suresh Sukumar

**Affiliations:** 1 Department of Medical Imaging Technology, Manipal College of Health Professions, Manipal Academy of Higher Education, Manipal, Karnataka, 576104, India; 2 Department of Exercise and Sports Sciences, Manipal College of Health Professions, Manipal Academy of Higher Education, Manipal, Karnataka, 576104, India; 3 Department of Radiodiagnosis and Imaging, Kasturba Medical College,, Manipal Academy of Higher Education, Manipal, Karnataka, India; 4 Dept. of Medical Diagnostic Imaging, College of Health Sciences, University of Sharjah, Sharjah, United Arab Emirates; 5 Dept. of Occupational Therapy, Manipal University College Malaysia, Meleka, Malaysia; 6 Dept. of Occupational Therapy, Manipal College of Health Professions, Manipal Academy of Higher Education, Manipal, Karnataka, India

**Keywords:** Sedentary behavior; Muscle thickness; Diaphragm; Physical activity; Ultrasonography

## Abstract

**Background:** The association between exercise and muscle build-up is a long-run connection. Whereas limited physical activity doesn’t do well with muscle build-up. But how much is that difference in muscle thickness between different levels of physical activity? **Aim:** To understand this we conducted a cross-sectional study to associate physical activity and sitting time with the muscle thickness of the lower limb and diaphragm. **Methods**: Patients ranging from 18-35 were chosen for this study. After enquiring about lifestyle factors like -smoking and drinking, out of 91 patients 30 patients smoke regularly and 6 patients had drinking habits. Also regarding occupation, 74.7% were employed and 25% were unemployed. We conducted this study on 91 participants who were grouped based on self-reported physical activity and sitting time levels based on IPAQ scores and underwent ultrasonography for quadriceps (rectus femoris and vastus intermedialis), soleus muscle, and diaphragm. **Results:** We found that the lower limb muscles have shown statistically significant differences between vigorous physical activity (VPA) and lower physical activity (LPA). We found that the quadriceps muscle(rectus femoris and vastus intermedialis) thickness was 1.3 cm in LPA whereas 2.8 cm in VPA with (p=<0.001) soleus muscle thickness being 1 cm in LPA and 2.2 cm. **Conclusions:** Physical activity levels are found to be positively related to the peripheral muscle thickness VPA (p=<0.001). Physical activity levels are found to be positively related to peripheral muscle thickness.

## Introduction

Physical activity (PA) (any bodily movement produced by skeletal muscles that require energy expenditure) is crucial for potential health benefits and protection against chronic diseases. ^1^ Insufficient physical activity and sedentary behavior (SB) (any waking behavior characterized by an energy expenditure of 1.5 metabolic equivalents (METS) or less while sitting or reclining) are now associated with an increased risk of cardiometabolic disease and cancer. ^2^ Experimental studies have administered several interventions to address the increasing burden of physical inactivity and SB. However, observational studies have established a relationship between PA and SB, with the health risks remaining still unclear, as there could be health risks associated with SB. ^3^

Muscle mass and strength are predictors of performance enhancement and ability to work in adults and mobility functions in the elderly population. Furthermore, peripheral muscle mass and strength are associated with chronic diseases like sarcopenia which is a major risk and early mortality. Though anecdotal evidence claims a bidirectional relationship between physical inactivity and peripheral muscle strength or thickness, observational studies establishing the relationship are lacking. In young, healthy people, there is a substantial correlation between overall muscular strength and higher-intensity PA, and age-related reductions in muscle size and strength have been seen to coincide with lower activity levels. ^4^^–^^6^ According to our knowledge, ^3^ a person’s level of moderate to vigorous physical activity (MVPA) is associated with broader benefits including improved cardiorespiratory fitness and total work capacity, but not directly to muscle growth and strength. ^4^^,^^7^ The evidence regarding the relationship between levels of PA and the peripheral muscle (soleus, gastrocnemius, and diaphragm) is still debatable using an ultrasonogram.

We hypothesized that: 1) there is a possible change in the muscle thickness and strength of individuals engaged in some PA compared to a sedentary lifestyle, and 2) a change in the thickness of muscles changes as age progresses. ^8^ Hence we aimed to relate various dimensions of PA and sitting time with the diaphragm & lower limb muscle thickness.

## Methods

### Study design

**Figure 1.**
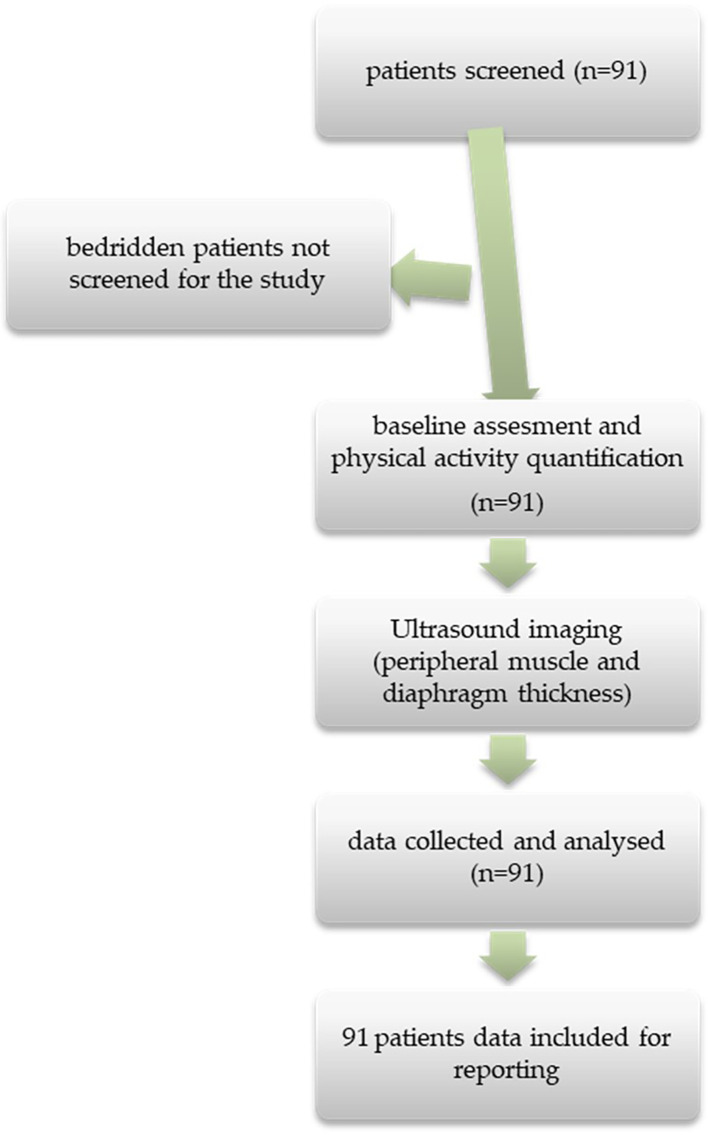
STROBE flow diagram showing the inclusion of participants.

This study was a prospective single-centered randomized crossover trial conducted between January 2022 to November 2022 in the Department of Radio-diagnosis and Imaging, Kasturba medical hospital, Manipal, India. Institutional Ethics Committee, KH (IEC2: 125/2022) and Clinical Trial Registry of India (CTRI/2022/10/046187) https://ctri.nic.in/Clinicaltrials/rmaindet.php?trialid=72850&EncHid=34225.86311&modid=1&compid=19 approved the study. Figure 1 depicts this methodology.

### Participants

We recruited the potential participants from the patients who were waiting for the radiological screening from the radiological department of the multidisciplinary teaching hospital. The written consent was obtained from the participants. The participants were first screened for the exclusion factors like recent trauma, orthopedic interventions, bed-ridden, paralyzed, osteoarthritis, and other chronic diseases of the heart and lungs which can hamper the diaphragm thickness. Hence, we included both male and female patients aged 18 – 35 years old for the following study.

### Physical activity

Self-reported PA was assessed using Short International Physical Activity Questionnaire (S-IPAQ) for young and middle-aged adults. The questionnaire evaluates the amount of time (frequency and duration) spent engaging in activities of vigorous, moderate intensity, walking, and sitting over the course of the previous seven days. The vigorous, moderate, and walking intensities were quantified as 8, 4, and 3.3 metabolic equivalents (METS).

*Sample size calculation*

We required 91 samples to achieve a moderate correlation (r1 > 0.4) at an alpha level of 95% and an 80% strength. The algorithm for determining the cumulative correlation coefficient distribution is used in all analyses. ^9^

### Muscle thickness

**Figure 2.**
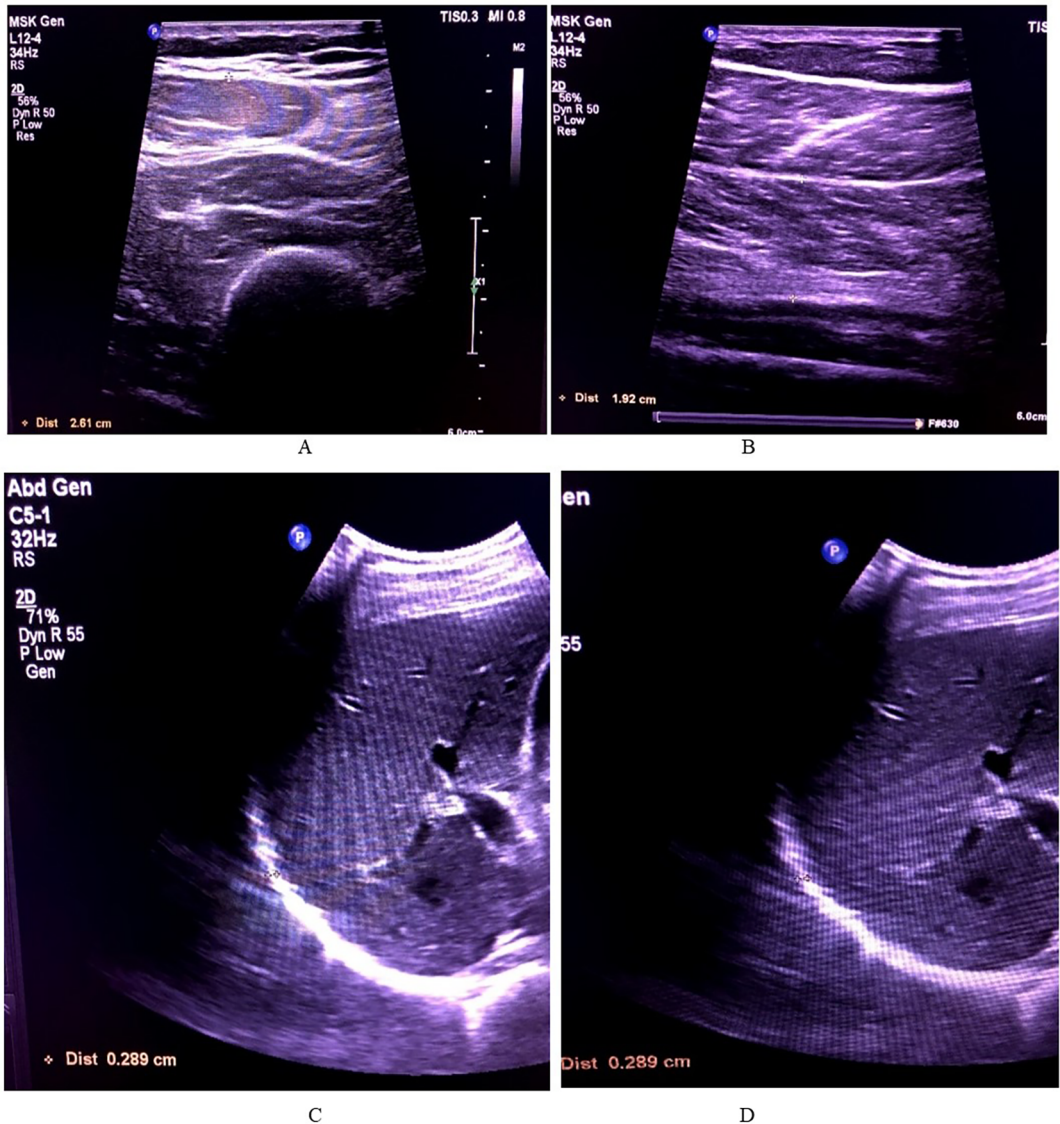
The thickness measurements of all the muscles. A – Soleus, B – Quadriceps (rectus femoris and vastus intermedialis), C – Diaphragm inhalation, D – Diaphragm exhalation.

The lower limb muscles measured in this study were the soleus and quadricep muscle (rectus femoris and vastus intermedialis) in both limbs. For measuring the diaphragm, the patient was laid supine and measured at both inhalation and exhalation using the M Mode ultrasonography. The measurement pattern is depicted in Figure 2

### Procedure

We used the ultrasound machine with linear and curvilinear transducers for the following study. All patients had to be screened for their anterior quadriceps, soleus, and diaphragm measurements.

### To measure the quadriceps

The anterior thigh muscle of all subjects will be measured using a 13MHz linear array probe. The B-model ultrasound was used to identify the anterior quadriceps muscle. The patient will be placed in a supine posture with their knees extended and their feet in a neutral position. The distance that lies between the anterior fascia of the rectus femoris muscle (RF) and the posterior fascia of the vastus intermedius muscle will be evaluated to calculate the anterior thigh muscle thickness (TMT). An axial cross-sectional image of the anterior quadricep muscle is obtained of both limbs and recorded. ^10^

• **To measure the soleus:**
  An ultrasound with a 13MHz linear probe was used on distal 1/3 of the calf length which was used to obtain soleus muscle imaging. The B-model ultrasound was used to identify the soleus muscle. Participants were oriented in a prone position, knees outstretched and 0° dorsiflexion of the ankle or knees bent at 30°, and ankle dorsiflexion at 0° in the prone position with a pillow underneath. To keep track of muscle movement, the ultrasound device was switched to M-mode to trace motion. ^11^
• **To measure the diaphragm:**
  The chest wall was aligned perpendicularly with a 13-MHz linear array transducer. Using M-mode, the diaphragmatic thickness was determined. Tdi, ee (Diaphragmatic thickness at end-expiration) and Tdi, pi (peak inspiration) measurements were already taken on consecutive breaths, which were seen in a single M-mode image. The diaphragmatic thickness was measured before normal inhalation and after complete exhalation.
  The thickness of the diaphragm for each experiment has been recorded. ^12^^,^^13^

## Results

The study included 91 patients aged 18 to 35 years with N = 78 male subjects with mean age and standard deviation of 27.954 years ± 4.67 and N = 13 females with mean age and standard deviation of 27.978 years ± 4.67

### Baseline characteristics

The research’s participants ranged in age from 18 to 35. Out of 91 patients, 30 were found to regularly smoke, and six had drinking habits after questions regarding lifestyle characteristics including smoking and drinking were asked. In terms of occupation, 74.7% of people had an occupation and 25% were unemployed. The following data is shown in Table 1.

**Table 1.** Demographic and various factors of the patients.

Variables		Mean ± SD	Number (%)
**Lifestyle**	Smoking	Chronic N = 30 [28.01 ± 4.509]	32.96
		Occasional N = 15 [27.61 ± 4.338]	16.48
		Nonsmoker N = 45 [27.9 ± 4.885]	49.45
	Alcohol	Chronic N = 6 [27.85 ± 3.109]	6.59
		Occasional N = 39 [27.90 ± 4.29]	42.85
		Nonalcoholic N = 45 [27.97 ± 4.67]	49.45
Occupation	Employed	N = 68 [27.9 ± 3.951]	74.7
	Unemployed	N = 23 [27.97 ± 3.514]	25
Physical activity levels	Vigorous	N = 39 [27.9 ± 4.375]	42.85
	Moderate	N = 46 [27.83 ± 4.749]	50.54
	Walking	N = 6 [27.77 ± 2.516]	6.59

### Physical activity among the participants

The participants were divided into three distinct categories: low (n = 6), intermediate (n = 46), and high METS score (n = 39, 42.85%). The results showed that the low METS score was 500.66 minutes per week, the moderate METS score was 1969.69 minutes per week, and the high METS score was 4408.17 minutes per week.

### Association between muscle thickness and physical activity

Based on the PA and IPAQ scores, we divided patients into low, moderate, and high PA. When we compared the muscle thickness with the PA, we found the following results. The left and right quadriceps values (rectus femoris and vastus intermedialis) were significantly increased as PA increased. The mean values were 1.3 cm for LPA, 1.7 cm for moderate PA, and 2.8 cm for VPA. Similarly, in the right and left soleus muscle thicknesses, the values increased from 1 cm for LPA to 1.56 cm for moderate PA and 2.2 cm for VPA. The diaphragm thickness showed an increase with PA ranging from 0.19 mm for LPA, 0.25 mm in moderate PA to 0.29 mm for VPA in full inhalation, compared to 0.18 mm, 0.23 mm, and 0.27 mm respectively for exhalation.

**Figure 3.**
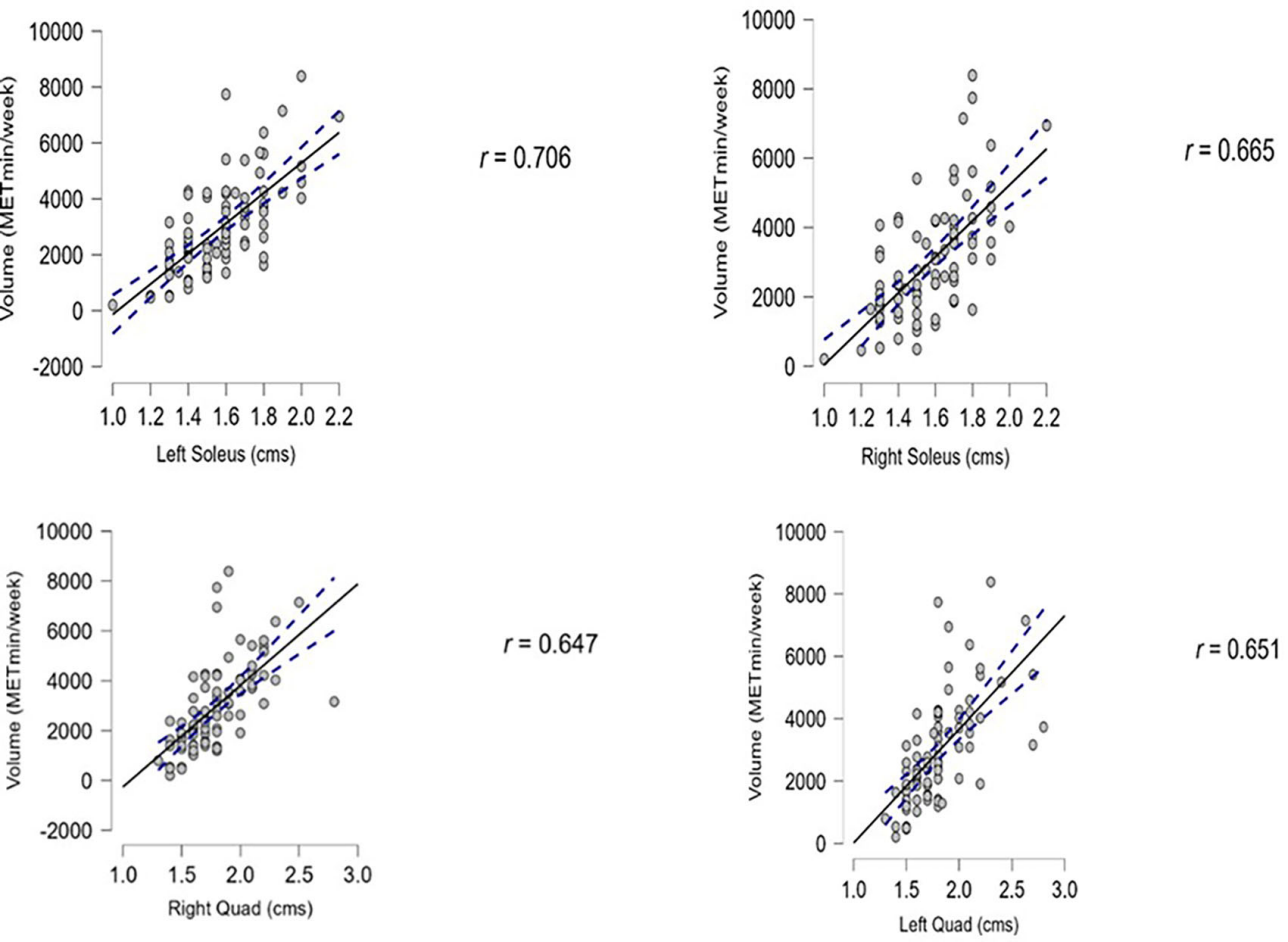
Shows the correlation between lower limb muscle thickness to the PA.

We found that the association between PA and muscle thickness was significant in the lower limb muscles, with a p-value lower than 0.01. The diaphragm thickness showed a positive association with PA but was not statistically significant, as the p-value were 0.35 for inhalation and 0.17 for exhalation. The data are presented in Table 2. The Pearson correlation results for lower limb muscle thickness with the PA levels are depicted in Figure 3. All the graphs depict a positive correlation between muscle thickness and PA ( Figure 3).

**Table 2.** Thickness values in comparison with PA.

Muscle	Low PA	Moderate PA	High PA	Volume (Met/Min/Week)	Pearson coefficient	p-value
Left quadriceps (rectus femoris and vastus intermedialis) (cm)	1.3	1.79	2.8	0.651	0.653	<0.001
Right quadriceps (rectus femoris and vastus intermedialis) (cm)	1.3	1.78	2.8	0.647	0.709	<0.001
Left soleus (cm)	1.0	1.56	2.2	0.706	0.68	<0.001
right soleus (cm)	1.0	1.55	2.2	0.665	0.646	<0.001
Inspiration diaphragm (mm)	0.19	0.25	0.29	0.057	0.097	0.358
Expiration diaphragm (mm)	0.18	0.23	0.27	-0.106	-0.143	0.178

MET – metabolic equivalent of task.

### Professional status and PA

Even if physical activity was based on patients’ self-report, the occupation made a difference. Desk-based workers mostly lead a sedentary lifestyle hence their PA level was comparatively lower than those who had an active lifestyle. ^14^ In the majority of the studies, unemployment is detrimental to health behavior. ^14^ Furthermore, it is believed that both the physical and social environments play an important role. In addition, Owen *et al.* reported that adult participation in PA was influenced by a range of personal, social, and environmental factors and those individual-level variables such as socioeconomic status and perceived self-efficacy demonstrated the strongest association with PA behavior (sitting time, workout time). ^14^^,^^15^

## Discussion

Our study aimed to look for the possible relationship between muscle thickness and various levels of PA. According to our research concept, the research was focused on a few factors, including age, appropriate muscles for this investigation, and potential repercussions. ^16^

### Physical activity levels in the participants

A total of 91 patients were included in our study, of which six were sorted into the LPA, N = 46 for moderate PA, and N = 39 for VPA; these make about 7% of the participants perform LPA, 50% with moderate PA, and 43% with VPA. Previous studies that have considered a larger population in India have found that around 54% of the total sample they had were physically inactive, and 14% had high PA. ^17^ Internationally, around 15.8% of the people in East and Southeast Asia are physically inactive. ^18^

### Muscle thickness in the participants

In our study, we observed that the soleus muscle thickness was 1 cm in LPA and 2.2 cm in VPA (p = 0.001), while the quadriceps muscle thickness (rectus femoris and vastus intermedialis) was 1.3 cm in LPA and 2.8 cm in VPA. The diaphragm thickness was 0.19 cm in LPA and 0.29 (p = 0.358) in PA. A study by Schoenfeld observed the difference in the muscle thickness for low *versus* high resistance exercises and found that the high resistance exercises were improving the quadriceps muscle thickness by 9.5%. ^19^ This supports our results that show that increased PA improves muscle thickness.

The study conducted by Silva *et al*. in 2010 observed that Asians have lower skeletal muscle mass as compared to African Americans, Whites, and Hispanics. ^20^ The muscle thickness that we measured in our study without considering the PA level was 1.78 cm and 1.79 cm for the right and left quadriceps (rectus femoris and vastus intermedialis) respectively, and 1.55 cm and 1.56 cm for the right and left soleus muscles respectively.

The reason for selecting the quadriceps, soleus in the lower limb, and the diaphragm for the study were that many researchers have found that there is a change in muscle thickness as age progresses, and it differs with sex as well. In 2010 Katsuo Fujiwara *et al.* reported that compared to their contemporaries in their 20s, men and women who were at least 60 years old had significantly thinner gastrocnemius muscles. With regards to the soleus, neither sex’s age group showed any appreciable changes in soleus thickness. For the gastrocnemius but not the soleus, muscle thickness decreased more from age 40 to 79. These findings support the idea that the gastrocnemius deteriorates and atrophies more rapidly than the soleus. One of the variables that contribute to a decline in muscle strength is aging. Age generally results in a loss of muscle mass and strength. ^8^ According to previoys studies, men’s skeletal muscle degradation is correlated with age at about 27 years of age. ^12^ With this clause, we have restricted our study age group to between 18-35 years. The diaphragm muscle thickness showed much less changes during inhalation and exhalation, which showed a negative association between inhalation and exhalation values. Enright *et al.* discovered that In healthy people, the dimensions of the diaphragm can be increased by weight training. The effect of inspiratory muscle training (IMT) on diaphragm thickness has not been previously reported in healthy people. ^21^ In Enright *et al.*’s study the group demonstrated an increase in diaphragm thickness. This increase in diaphragm thickness may result in increased inspiratory muscle efficiency or improved pulmonary mechanics, or both.

In this study, we focused on the lower limb muscles and diaphragm to get a prospective idea of the relationship of these muscles with PA. When humans are physically active, the lower body is most engaged in these activities. PA could be as simple as walking or running. Most likely, the lower body muscles are active while the breathing pattern changes simultaneously, therefore the diaphragm is engaged too. Recent studies have shown and proved that diaphragm muscle thickness changes with increased PA, *e.g.* weight training. ^21^ In addition, quadriceps, soleus, and gastrocnemius muscles show the greatest activation during the quiet standing posture, These muscles are also vigorously activated in the stance phase of walking to maintain the standing posture and generate forces for propulsion.

With all these factors as constants and variables, our study shows that with an increase in PA, there is a significant increase in the quadriceps (rectus femoris and vastus intermedialis), soleus muscle and diaphragm thicknesses, with mean values of 1.3 cm, 1.78 cm and 2.8 cm in LPA, moderate PA and VPA respectively for the quadriceps muscle (rectus femoris and vastus intermedialis); 1 cm, 1.56 cm and 2.2 cm for soleus, and 0.19 mm, 0.25 mm and 0.29 mm for the diaphragm, with increasing PA levels from LPA to moderate PA to VPA respectively. The changes in the lower limbs showed statistically significant results.

## Conclusions

Peripheral muscle thickness has been found to positively correlate with physical activity levels. However future trials should further expand the association with the objectively measured PA levels.

### Limitations and recommendations

Due to the fact that the PA measures utilized in the study were self-reported, there is a risk of recalling bias and response bias. Instead of employing a self-reported questionnaire, future studies could use objectively assessed PA.

Using IPAQ, which provides subjective measurement, we were able to determine the patients’ PA parameters in the current study. Due to observational studies’ use of self-perceived PA, which is frequently unjustified, our comprehension of the association between PA and muscle thickness currently is still unclear. This calls for additional studies employing objectively measured PA.

The nature of the cross-sectional approach used in the research made it difficult to determine the actual link between PA and changes in muscle thickness. If one adopts this approach, one might have a better grasp of how lifestyle factors affect individual muscle strength. Future research should look into these lifestyle choices and take them into account since they can have an impact on these results. Understanding how PA and lifestyle choices affect muscular strength requires studies that demonstrate associations between changes in muscle thickness and PA.

## Data Availability

Harvard Dataverse: Active adults have thicker peripheral muscles and diaphragm: a cross-sectional study, https://doi.org/10.7910/DVN/MVFLMY. This project contains the following underlying data:
- Aishwarya data sheet.xlsx
