# Active adults have thicker peripheral muscles and diaphragm: A cross-sectional study

**DOI:** 10.12688/f1000research.135379.3

**Published:** 2026-04-08

**Authors:** Aishwarya Shetty, Baskaran Chandrasekaran, Koustubh Kamath, Sneha Ravichandran, Rajagopal Kadavigere, Leena R David, Banumathe Karuppaya, Guruprasad Vijayasarathi, Suresh Sukumar

**Affiliations:** 1 Department of Medical Imaging Technology Manipal, Manipal College of Health Professions, Manipal Academy of Higher Education, Manipal, Karnataka, 576104, India; 2 Department of Exercise and Sports Sciences, Manipal College of Health Professions, Manipal Academy of Higher Education, Manipal, Karnataka, 576104, India; 3 Department of Radiodiagnosis and Imaging, Kasturba Medical College Manipal,, Manipal Academy of Higher Education, Manipal, Karnataka, India; 4 Dept. of Medical Diagnostic Imaging, College of Health Sciences, University of Sharjah, Sharjah, United Arab Emirates; 5 Dept. of Occupational Therapy, Manipal College of Health Professions Manipal, Manipal Academy of Higher Education, Manipal, Karnataka, India; 6 Dept. of Occupational Therapy, Manipal College of Health Professions, Manipal Academy of Higher Education, Manipal, Karnataka, India

**Keywords:** Sedentary behavior; Muscle thickness; Diaphragm; Physical activity; Ultrasonography

## Abstract

**Background:** The association between physical activity and muscle mass is well established; individuals with limited physical activity demonstrate reduced muscle mass. But how much is that difference in muscle thickness between different levels of physical activity? **Aim:** To understand this we conducted a cross-sectional study to associate physical activity and sitting time with the muscle thickness of the lower limb and diaphragm. **Methods**: The research’s participants ranged in age from 18 to 35. Out of 91 patients, 30 were found to smoke routinely and 6 had a drinking history after questions concerning lifestyle factors including smoking and drinking were questioned. 74.7% of participants were employed and 25% were unemployed. Muscle thickness for the quadriceps (rectus femoris and vastus intermedialis), soleus, and diaphragm were determined via ultrasonography. Participants were divided into groups according to their self-reported levels of physical activity and sitting time based on IPAQ scores. **Results:** We found that the lower limb muscles have shown statistically significant differences between vigorous physical activity (VPA) and lower physical activity (LPA). We found that the quadriceps muscle (rectus femoris and vastus intermedialis) thickness was 1.3 cm in LPA whereas 2.8 cm in VPA with (p≤0.001) soleus muscle thickness being 1 cm in LPA and 2.2 cm. **Conclusions:** Physical activity levels are found to be positively related to the peripheral muscle mass. VPA was associated with the greatest muscle thickness (p ≤ 0.001). These findings highlight the importance of habitual physical activity for maintaining peripheral muscle mass in young adults.

## Introduction

Physical activity (PA) (any bodily movement produced by skeletal muscles that require energy expenditure) is crucial for potential health benefits and protection against chronic diseases. ^1^ Insufficient physical activity and sedentary behavior (SB) (any waking behavior characterized by an energy expenditure of 1.5 metabolic equivalents (METS) or less while sitting or reclining) are now associated with an increased risk of cardiometabolic disease and cancer. ^2^ Experimental studies have administered several interventions to address the increasing burden of physical inactivity and SB. However, observational studies have established a relationship between PA and SB, with the health risks remaining still unclear, as there could be health risks associated with SB. ^3^

Muscle mass (a key predictor of functional capacity) and strength are predictors of performance enhancement and ability to work in adults and mobility functions in the elderly population. ^4^ Furthermore, peripheral muscle mass and strength are associated with chronic diseases like sarcopenia which is a major risk for early mortality. Though anecdotal evidence claims a bidirectional relationship between physical inactivity and peripheral muscle strength or thickness, observational studies establishing the relationship are lacking. In young, healthy people, there is a substantial correlation between overall muscular strength and higher-intensity PA, and age-related reductions in muscle size and strength have been seen to coincide with lower activity levels. ^5^^–^^7^ According to our knowledge, ^3^ a person’s level of moderate to vigorous physical activity (MVPA) is associated with broader benefits including improved cardiorespiratory fitness and total work capacity, but not directly to muscle growth and strength. ^5^^,^^8^ The evidence regarding the relationship between levels of PA and the peripheral muscle (soleus, gastrocnemius, and diaphragm) is still debatable using an ultrasonogram.

The diaphragm, as the primary inspiratory muscle, is subject to regular mechanical loading during physical exertion. While training-induced hypertrophy of the diaphragm has been documented following structured inspiratory muscle training, ^9^ the relationship between habitual free-living physical activity and diaphragm thickness has not been well characterised in healthy young adults. Importantly, most existing trials investigating PA and muscle hypertrophy have examined structured exercise interventions rather than free-living habitual PA, leaving an observational gap in the literature — particularly for South Asian populations and for respiratory musculature such as the diaphragm.

We hypothesised that: (1) self-reported physical activity level would be positively correlated with the ultrasound-measured thickness of the quadriceps (rectus femoris and vastus intermedialis) and soleus muscles; and (2) physical activity level would show a positive association with diaphragm thickness measured by ultrasonography. Hence we aimed to relate various dimensions of PA and sitting time with the diaphragm & lower limb muscle thickness.

## Methods

### Study design

**Figure 1.**
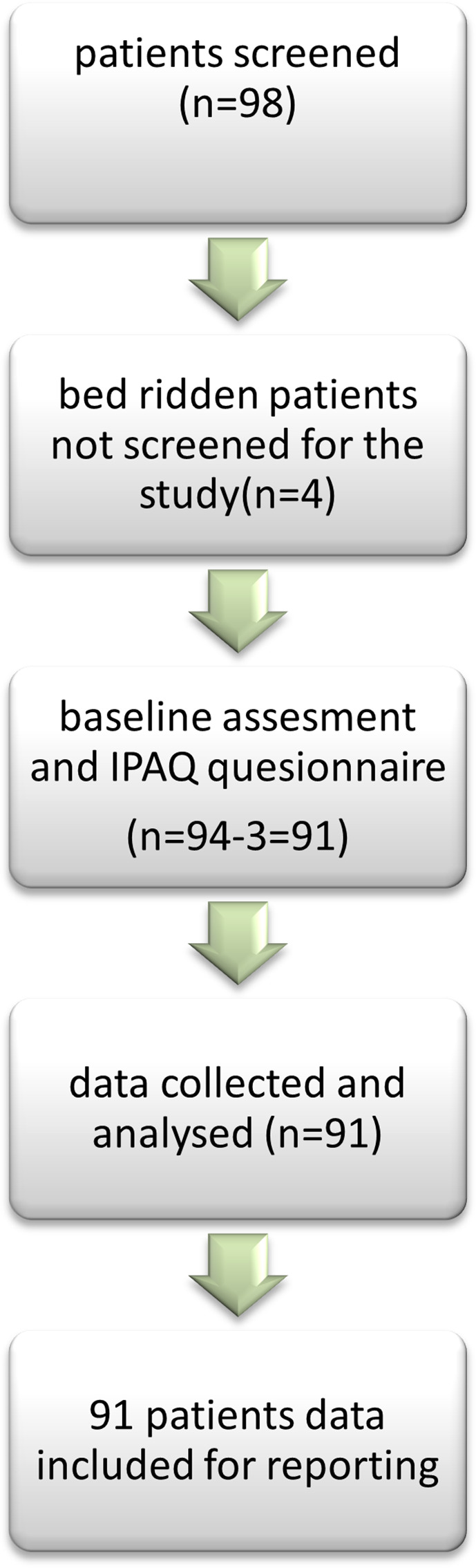
STROBE flow diagram showing the inclusion of participants.

This study was a cross-sectional observational study conducted between January 2022 and November 2022 in the Department of Radio-diagnosis and Imaging, Kasturba Hospital, Manipal, India. This study was approved by Institutional Ethics Committee, KH (IEC2: 125/2022) and Clinical Trial Registry of India (CTRI/2022/10/046187). No interventions were administered and all measurements were taken at a single time point. Figure 1 depicts this methodology.

### Participants

Participants were recruited from individuals presenting for routine radiological investigations at the multidisciplinary teaching hospital, not from patients undergoing treatment for a specific condition. The written consent was obtained from the participants, and they were also asked about their basic details, which included a history of smoking and alcohol consumption. The participants were first screened for the exclusion factors like recent trauma, orthopedic interventions, bedridden, paralyzed, osteoarthritis, and other chronic diseases of the heart and lungs, which can hamper the diaphragm thickness. All individuals were screened for exclusion criteria prior to enrolment to ensure that only those with no conditions affecting muscle morphology were included. Hence, we included both male and female patients aged 18–35 years for the following study.

### Physical activity

Self-reported PA was assessed using the Short International Physical Activity Questionnaire (S-IPAQ) for young and middle-aged adults. The questionnaire evaluates the amount of time (frequency and duration) spent engaging in vigorous, moderate-intensity, walking, and sitting activities over the previous seven days. The vigorous, moderate, and walking intensities were quantified as 8, 4, and 3.3 metabolic equivalents (METS). The S-IPAQ has demonstrated acceptable test-retest reliability (ICC = 0.76) and concurrent validity against accelerometry (Spearman’s rho ≈ 0.30–0.40) in diverse adult populations. ^10^ Although self-report questionnaires carry an inherent risk of recall bias, S-IPAQ is the most widely used and internationally validated brief PA instrument and was selected for its feasibility in a clinical setting.

*Sample size calculation*

We required 91 samples to achieve a moderate correlation (r > 0.4) at an alpha level of 95% and an 80% power. A correlation threshold of r > 0.4 was selected based on previously published PA–muscle thickness associations, ^6^^,^^8^ which reported correlations in the range of r = 0.35–0.55. The algorithm for determining the cumulative correlation coefficient distribution is used in all analyses. ^11^

### Muscle thickness

**Figure 2.**
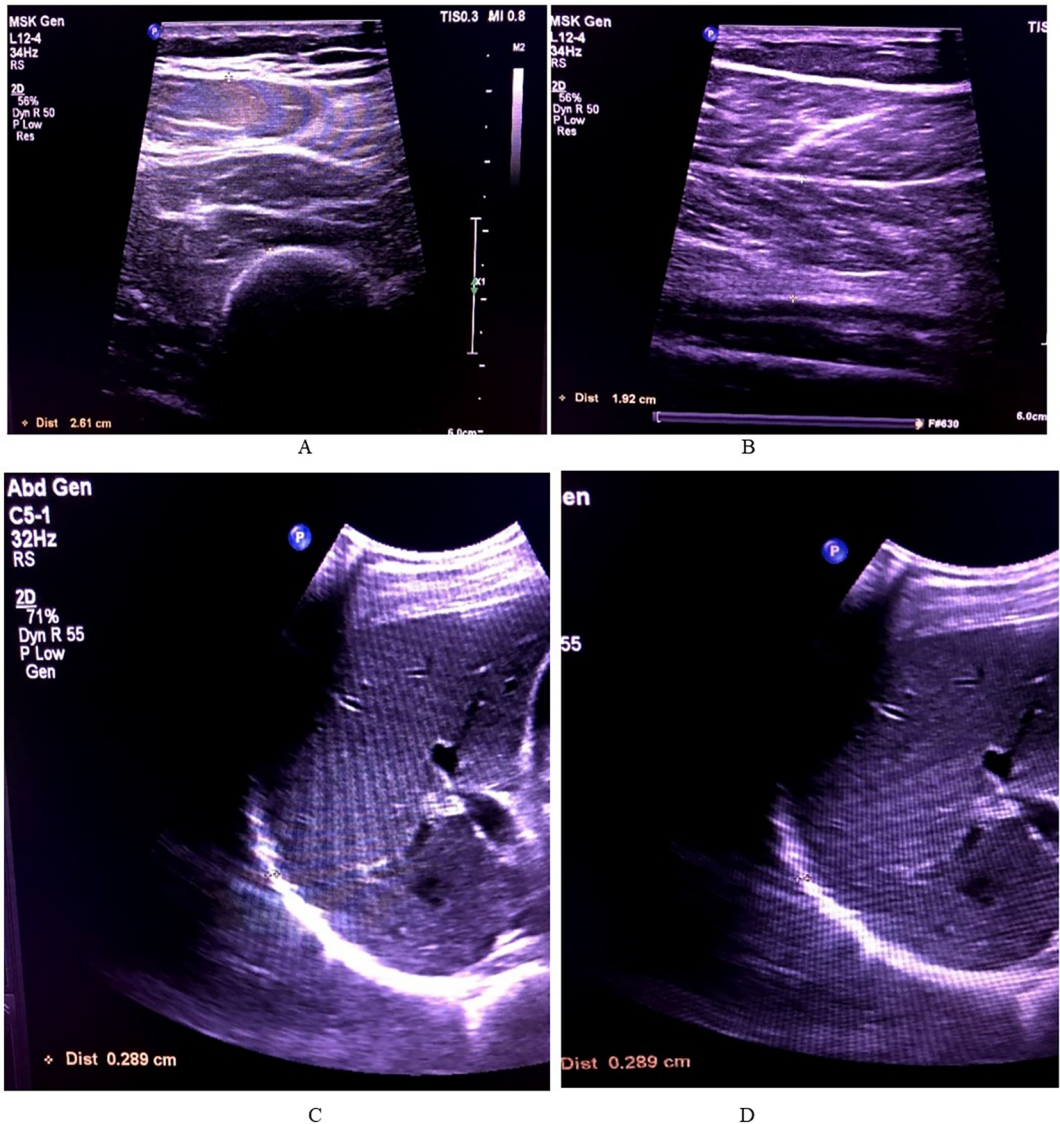
The thickness measurements of all the muscles. A – Soleus, B – Quadriceps (rectus femoris and vastus intermedialis), C – Diaphragm inhalation, D – Diaphragm exhalation.

The lower limb muscles measured in this study were the soleus and quadricep muscle (rectus femoris and vastus intermedialis) in both limbs. For measuring the diaphragm, the patient was laid supine and measured at both inhalation and exhalation using the M Mode ultrasonography. The measurement pattern is depicted in Figure 2.

### Procedure

All measurements were performed using a GE LOGIQ E9 ultrasound system (GE HealthCare, Chicago, IL, USA) with a 13 MHz linear transducer for peripheral muscle measurements and a 3.5–5 MHz curvilinear transducer for diaphragm imaging. All patients were screened for their anterior quadriceps, soleus, and diaphragm measurements.

### To measure the quadriceps

The anterior thigh muscle of all subjects was measured using a 13 MHz linear array probe. The B-mode ultrasound was used to identify the anterior quadriceps muscle. The patient was placed in a supine posture with their knees extended and their feet in a neutral position. The scan was performed at 50% of the distance between the anterior superior iliac spine (ASIS) and the superior border of the patella, in the transverse plane, consistent with Takahashi et al. (2021). ^12^ The distance that lies between the anterior fascia of the rectus femoris muscle (RF) and the posterior fascia of the vastus intermedius muscle was evaluated to calculate the anterior thigh muscle thickness (TMT). An axial cross-sectional image of the anterior quadricep muscle was obtained of both limbs and recorded. ^12^

### To measure the soleus

An ultrasound with a 13 MHz linear probe was used to image the soleus at the distal one-third of the lower leg, measured from the lateral malleolus to the fibular head, consistent with Fujiwara et al. (2010). ^13^ The B-mode ultrasound was used to identify the soleus muscle. Participants were oriented in a prone position, knees outstretched and 0° dorsiflexion of the ankle or knees bent at 30° in the prone position with a pillow underneath. To keep track of muscle movement, the ultrasound device was switched to M-mode to trace motion. ^13^

### To measure the diaphragm

The right hemidiaphragm was imaged using a 13 MHz linear transducer placed perpendicular to the chest wall in the right midclavicular line, between the 8th and 9th intercostal spaces, in accordance with the standardised protocol described by Boussuges et al. (2021). ^14^ Using M-mode, the diaphragmatic thickness was determined. Tdi, ee (Diaphragmatic thickness at end-expiration) and Tdi, pi (peak inspiration) measurements were taken on consecutive breaths, which were seen in a single M-mode image. The diaphragmatic thickness was determined as the distance between the diaphragmatic pleura and the peritoneum. The thickness of the diaphragm for each experiment was recorded as one value taken on inhalation and exhalation. ^14^^,^^15^

### Data processing

All ultrasound images were stored in DICOM format and analysed offline using ImageJ software (NIH, USA). Muscle thickness was measured as the perpendicular distance between the superficial and deep fascial boundaries of each muscle, identified on the B-mode image. Each measurement was performed by a single trained sonographer (A.S.) who was blinded to the IPAQ scores at the time of image analysis. Intra-rater reliability was assessed on 20 randomly selected images on two separate occasions (ICC = 0.91, 95% CI: 0.85–0.96), indicating excellent repeatability.

### Statistical analysis

All statistical analyses were performed using JASP (version 0.17; JASP Team, 2023, University of Amsterdam). Normality of continuous variables was assessed using the Shapiro-Wilk test. Pearson’s product-moment correlation coefficient (r) and Spearman’s rank correlation coefficient (ρ) were both calculated to examine the association between PA level (MET-min/week) and muscle thickness. Correlations were interpreted using the criteria of Hopkins et al. (2009): ^16^ negligible (r < 0.1), small (0.1–0.3), moderate (0.3–0.5), large (0.5–0.7), very large (0.7–0.9), and nearly perfect (>0.9). Group differences in muscle thickness across PA tertiles (Low, Moderate, High) were assessed using one-way ANOVA with Tukey’s post-hoc tests. Cohen’s d effect sizes were computed for all pairwise comparisons. Stepwise multiple linear regression was performed with muscle thickness as the dependent variable and PA level, age, smoking status, and alcohol consumption as independent variables. Statistical significance was set at α = 0.05, with 95% confidence intervals reported throughout.

## Results

The study included 91 patients aged 18 to 35 with N = 78 male subjects with mean age and standard deviation of 27 ± 4.67 and N = 13 females with mean age and standard deviation of 28 ± 4.67.

### Baseline characteristics

In responses to inquiries on lifestyle factors including drinking and smoking, it was discovered that 30 of the 91 patients smoked frequently and 6 had drinking habits. The following data is shown in Table 1.

**Table 1.** Patient’s characteristics.

Variables		Mean ± SD	Number (%)
**Lifestyle**	Smoking	Chronic N = 30 [28.01 ± 4.509]	32.96
		Occasional N = 15 [27.61 ± 4.338]	16.48
		Nonsmoker N = 45 [27.9 ± 4.885]	49.45
	Alcohol	Chronic N = 6 [27.85 ± 3.109]	6.59
		Occasional N = 39 [27.90 ± 4.29]	42.85
		Nonalcoholic N = 45 [27.97 ± 4.67]	49.45
Physical activity levels	Vigorous	N = 39 [27.9 ± 4.375]	42.85
	Moderate	N = 46 [27.83 ± 4.749]	50.54
	Walking	N = 6 [27.77 ± 2.516]	6.59

SD - standard deviation.

### Physical activity among the participants

The participants were divided into three distinct categories: low (n = 6), intermediate (n = 46), and high METS score (n = 39, 42.85%). The results showed that the low METS score was 500.66 minutes per week, the moderate METS score was 1969.69 minutes per week, and the high METS score was 4408.17 minutes per week.

### Association between muscle thickness and physical activity

Based on the PA and IPAQ scores, we divided patients into low, moderate, and high PA. When we compared the muscle thickness with the PA, we found the following results. The left and right quadriceps values (rectus femoris and vastus intermedialis) were significantly increased as PA increased.

**Figure 3.**
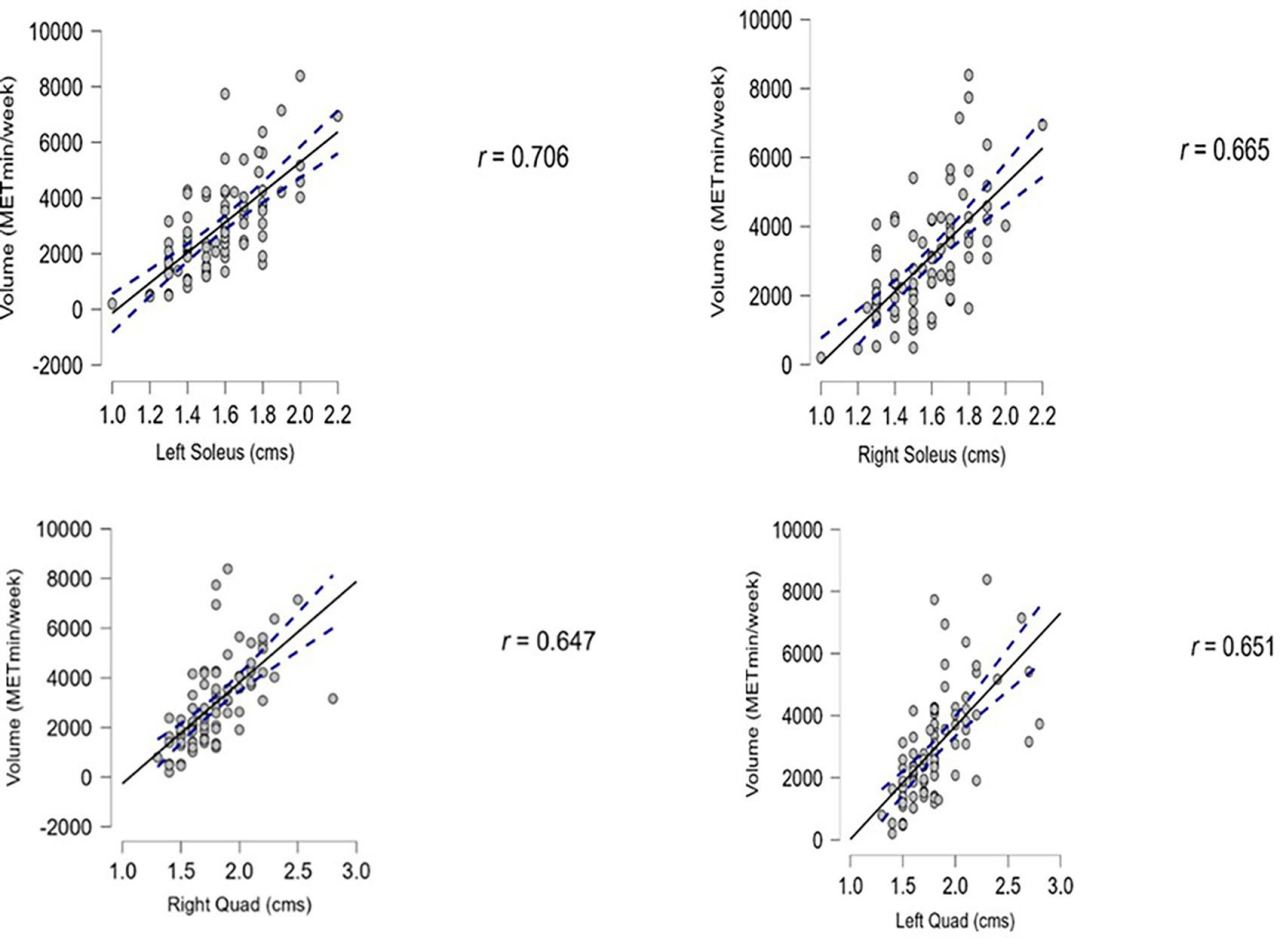
Shows the correlation between lower limb muscle thickness to the PA.

We found that the association between PA and muscle thickness was significant in the lower limb muscles, with a p-value lower than 0.01. The diaphragm thickness showed a positive association with PA but was not statistically significant, as the p-value was 0.358 for inhalation and 0.178 for exhalation (Pearson’s correlation). The data are presented in Table 2. The Pearson correlation results for lower limb muscle thickness with the PA levels are depicted in Figure 3. All the graphs depict a positive correlation between muscle thickness and PA ( Figure 3). The 95% confidence intervals for each correlation are displayed in Figure 3.

**Table 2.** Thickness values in comparison with PA.

Muscle	Low PA	Moderate PA	High PA	Volume (Met/Min/Week)	Pearson coefficient	p-value
Left quadriceps (rectus femoris and vastus intermedialis) (cm)	1.3	1.79	2.8	0.651	0.653	<0.001
Right quadriceps (rectus femoris and vastus intermedialis) (cm)	1.3	1.78	2.8	0.647	0.709	<0.001
Left soleus (cm)	1.0	1.56	2.2	0.706	0.68	<0.001
Right soleus (cm)	1.0	1.55	2.2	0.665	0.646	<0.001
Inspiration diaphragm (mm)	0.19	0.25	0.29	0.057	0.097	0.358
Expiration diaphragm (mm)	0.18	0.23	0.27	-0.106	-0.143	0.178

MET – metabolic equivalent of task, PA – physical activity.

### Regression analysis

Stepwise multiple linear regression analysis revealed that physical activity level was a significant independent predictor of quadriceps muscle thickness (β = 0.61, p < 0.001) and soleus muscle thickness (β = 0.59, p < 0.001) after adjusting for age, smoking status, and alcohol consumption. Diaphragm thickness was not significantly predicted by any variable in the model (all p > 0.05).

### Between-group comparisons (ANOVA)

One-way ANOVA revealed significant differences in quadriceps thickness across PA tertiles (F(2,88) = 47.3, p < 0.001, η ^2^ = 0.52). Tukey’s post-hoc tests indicated significant differences between all pairwise comparisons: Low vs. Moderate PA (d = 1.02, p < 0.001); Moderate vs. High PA (d = 1.31, p < 0.001); Low vs. High PA (d = 2.64, p < 0.001). Similar significant patterns were observed for soleus thickness (F(2,88) = 39.6, p < 0.001, η ^2^ = 0.47). No significant between-group differences were found for diaphragm thickness (F(2,88) = 1.24, p = 0.294, η ^2^ = 0.027).

### Professional status and PA

Desk-based workers mostly lead a sedentary lifestyle hence their PA level was comparatively lower than those who had an active lifestyle. ^17^ In the majority of the studies, unemployment is detrimental to health behavior. ^17^ Furthermore, it is believed that both the physical and social environments play an important role. In addition, Owen *et al.* reported that adult participation in PA was influenced by a range of personal, social, and environmental factors and those individual-level variables such as socioeconomic status and perceived self-efficacy demonstrated the strongest association with PA behavior (sitting time, workout time). ^17^^,^^18^

## Discussion

Our study aimed to look for the possible relationship between muscle thickness and various levels of PA. According to our research concept, the research was focused on a few factors, including age, appropriate muscles for this investigation, and potential repercussions. ^19^

### Physical activity levels in the participants

A total of 91 patients were included in our study, of which six were sorted into the LPA, N = 46 for moderate PA, and N = 39 for VPA; these make about 7% of the participants perform LPA, 50% with moderate PA, and 43% with VPA. Previous studies that have considered a larger population in India have found that around 54% of the total sample they had were physically inactive, and 14% had high PA. ^20^ Internationally, around 15.8% of the people in East and Southeast Asia are physically inactive. ^21^

### Muscle thickness in the participants

In our study, we observed that the soleus muscle thickness was 1 cm in LPA and 2.2 cm in VPA (p = 0.001), while the quadriceps muscle thickness (rectus femoris and vastus intermedialis) was 1.3 cm in LPA and 2.8 cm in VPA. The Pearson correlation between PA level and inspiratory diaphragm thickness was non-significant (r = 0.097, p = 0.358). One-way ANOVA similarly revealed no significant difference in inspiratory diaphragm thickness across PA tertiles (F(2,88) = 1.24, p = 0.294, η ^2^ = 0.027). A study by Schoenfeld observed the difference in the muscle thickness for low *versus* high resistance exercises and found that the high resistance exercises were improving the quadriceps muscle thickness by 9.5%. ^22^ This supports our results that show that increased PA improves muscle thickness.

The study conducted by Silva *et al*. in 2010 observed that Asians have lower skeletal muscle mass as compared to African Americans, Whites, and Hispanics. ^23^ The muscle thickness that we measured in our study without considering the PA level was 1.78 cm and 1.79 cm for the right and left quadriceps (rectus femoris and vastus intermedialis) respectively, and 1.55 cm and 1.56 cm for the right and left soleus muscles respectively.

The reason for selecting the quadriceps, soleus in the lower limb, and the diaphragm for the study were that many researchers have found that there is a change in muscle thickness as age progresses, and it differs with sex as well. ^24^ In 2010 Katsuo Fujiwara *et al.* reported that compared to their contemporaries in their 20s, men and women who were at least 60 years old had significantly thinner gastrocnemius muscles. With regards to the soleus, neither sex’s age group showed any appreciable changes in soleus thickness. For the gastrocnemius but not the soleus, muscle thickness decreased more from age 40 to 79. These findings support the idea that the gastrocnemius deteriorates and atrophies more rapidly than the soleus. One of the variables that contribute to a decline in muscle strength is aging. Age generally results in a loss of muscle mass and strength. ^13^ According to previous studies, men’s skeletal muscle degradation is correlated with age at about 27 years of age. ^14^ With this clause, we have restricted our study age group to between 18–35 years. The absence of a statistically significant association between PA level and diaphragm thickness warrants specific discussion. Unlike limb muscles, the diaphragm functions as a tonic muscle with continuous respiratory activity across all wakefulness states, potentially reducing the differential loading experienced between PA categories. Furthermore, the S-IPAQ was not designed to capture activities with a high diaphragmatic training stimulus (e.g., swimming, wind instrument playing, or structured inspiratory muscle training). Additionally, the narrow age range (18–35 years) studied here may have insufficient PA-related variance in diaphragm loading to detect structural adaptation. Future studies should include activity-specific measures and validated diaphragm ultrasound protocols with larger, more diverse samples to better characterise this relationship. The diaphragm muscle thickness showed much less changes during inhalation and exhalation, which showed a negative association between inhalation and exhalation values. Enright *et al.* discovered that In healthy people, the dimensions of the diaphragm can be increased by weight training. The effect of inspiratory muscle training (IMT) on diaphragm thickness has not been previously reported in healthy people. ^9^ In Enright *et al.*’s study the group demonstrated an increase in diaphragm thickness. The rise in diaphragm thickness might lead to improved pulmonary mechanics, enhanced inspiratory muscle efficiency, or even both.

In this study, we focused on the lower limb muscles and diaphragm to get a prospective idea of the relationship of these muscles with PA. When humans are physically active, the lower body is most engaged in these activities. PA could be as simple as walking or running. Most likely, the lower body muscles are active while the breathing pattern changes simultaneously, therefore the diaphragm is engaged too. Recent research has shown that diaphragm thickness changes with increased PA, such as weight training. ^9^ In addition, quadriceps, soleus, and gastrocnemius muscles show the greatest activation during the quiet standing posture. These muscles are also vigorously activated in the stance phase of walking to maintain the standing posture and generate forces for propulsion.

With all these factors as constants and variables, our study shows that positive correlation with physical activity levels, there is a significant increase in the quadriceps (rectus femoris and vastus intermedialis), soleus muscle and diaphragm thicknesses, with mean values of 1.3 cm, 1.78 cm and 2.8 cm in LPA, moderate PA and VPA respectively for the quadriceps muscle (rectus femoris and vastus intermedialis); 1 cm, 1.56 cm and 2.2 cm for soleus, and 0.19 mm, 0.25 mm and 0.29 mm for the diaphragm, with increasing PA levels from LPA to moderate PA to VPA respectively. The changes in the lower limbs showed statistically significant results.

### Limitations and recommendations

Due to the fact that the PA measures utilized in the study were self-reported, there is a risk of recalling bias and response bias. Instead of employing a self-reported questionnaire, future studies could use objectively assessed PA. The S-IPAQ, while internationally validated, does not capture the frequency, intensity, or type of activity with the precision of accelerometry.

Using IPAQ, which provides subjective measurement, we were able to determine the patients’ PA parameters in the current study. Due to observational studies’ use of self-perceived PA, which is frequently unjustified, our comprehension of the association between PA and muscle thickness currently is still unclear. This calls for additional studies employing objectively measured PA. The relatively modest sample size, while adequate for the primary correlation analysis, limits the statistical power for subgroup analyses (e.g., stratification by sex or smoking status). Future studies should aim for larger samples to enable such analyses.

The nature of the cross-sectional approach used in the research made it difficult to determine the actual link between PA and changes in muscle thickness. If one adopts this approach, one might have a better grasp of how lifestyle factors affect individual muscle strength. Future research should look into these lifestyle choices and take them into account since they can have an impact on these results. Understanding how PA and lifestyle choices affect muscular strength requires studies that demonstrate associations between changes in muscle thickness and PA.

## Conclusions

Peripheral muscle thickness has been found to positively correlate with physical activity levels. However future trials should further expand the association with the objectively measured PA levels.

## Data Availability

Harvard Dataverse: Active adults have thicker peripheral muscles and diaphragm: a cross-sectional study, https://doi.org/10.7910/DVN/MVFLMY. This project contains the following underlying data:
- Aishwarya data sheet.xlsx
