# Supplementary material for: Active adults have thicker peripheral muscles and diaphragm: A cross-sectional study
Source: F1000Res. 2026 Apr 8;12:836. Originally published 2023 Jul 17. [Version 3] doi: 10.12688/f1000research.135379.3 (PMC13062771; doi:10.12688/f1000research.135379.3)
Supplement: Peer review history

# Reviewer report: Reviewer response for version 3

**DOI:** 10.5256/f1000research.197522.r473982

**Published:** 2026-04-09

**Authors:** Dustin J Oranchuk

**Affiliations:** 1 Physical Medicine and Rehabilitation, University of Colorado Denver, Denver, Colorado, USA

The authors have done a great job of addressing my comments. While no study is perfect, this paper has been substantially improved.

 Well done.

Is the work clearly and accurately presented and does it cite the current literature?

No

If applicable, is the statistical analysis and its interpretation appropriate?

No

Are all the source data underlying the results available to ensure full reproducibility?

Yes

Is the study design appropriate and is the work technically sound?

Partly

Are the conclusions drawn adequately supported by the results?

Yes

Are sufficient details of methods and analysis provided to allow replication by others?

No

Reviewer Expertise:

Muscle morphology, physical performance, healthy ageing, strength and conditioning.

I confirm that I have read this submission and believe that I have an appropriate level of expertise to confirm that it is of an acceptable scientific standard.

# Reviewer report: Reviewer response for version 2

**DOI:** 10.5256/f1000research.155807.r242606

**Published:** 2024-09-04

**Authors:** Ramprasad Muthukrishnan, Renuka M

**Affiliations:** 1 Department of Physiotherapy, College of Health Science, Gulf Medical University, Ajman, United Arab Emirates; 2 Physiotherpy, Gulf Medical University, Ajman, United Arab Emirates

Good work,

 1. 'Active adults have thicker peripheral muscles and diaphragm' title can be re-written based on study aims and objectives. Is there a need of conclusive title really reflects world view? if conclusion drawn as study titles study methods need to be rigor and robust, such as RCT. 

 2. Why diaphragm is chosen along with peripheral muscles? is it convenient to do US scan? or any valid reasons? 

 3. Its mentioned that change in the thickness of muscles changes as age

 progresses, but its not tested age wise. If the age group is restricted between 18-35 this

 question being raised is appropriate?

 4. Method-Wide range of age with comorbidities included would give more insight or would have done on normal individuals. Provide clear insights with reasoner? 

 5. In abstract and in title its mentioned cross-sectional study but in methodology its mentioned as single-centred randomized crossover trial. Is the study RCT? later changed? if changed permission from IRB would elucidate the readers?

 6. Reasoning for no change in the diaphragm muscle is not discussed. Discussion would have provided more insights.

Is the work clearly and accurately presented and does it cite the current literature?

Yes

If applicable, is the statistical analysis and its interpretation appropriate?

I cannot comment. A qualified statistician is required.

Are all the source data underlying the results available to ensure full reproducibility?

Yes

Is the study design appropriate and is the work technically sound?

Yes

Are the conclusions drawn adequately supported by the results?

Yes

Are sufficient details of methods and analysis provided to allow replication by others?

Yes

Reviewer Expertise:

Back pain and motor control exercises, Neural correlates and executive functions, manual therapy

We confirm that we have read this submission and believe that we have an appropriate level of expertise to confirm that it is of an acceptable scientific standard, however we have significant reservations, as outlined above.

# Response

**Published:** 2026-03-07

**Authors:** suresh sukumar

**Affiliations:** Manipal College of Health Professions Manipal Academy of Higher Education, India

Good work,

 1)'Active adults have thicker peripheral muscles and diaphragm' title can be re-written based on study aims and objectives. Is there a need of conclusive title really reflects world view? if conclusion drawn as study titles study methods need to be rigor and robust, such as RCT.

**Response:** We acknowledge the reviewer's valid point regarding the conclusive nature of the title. However, it is common practice in observational research to use descriptive titles that summarise the primary finding, particularly in cross-sectional studies. We note that the subtitle "A cross-sectional study" explicitly communicates the observational design, setting appropriate expectations for the reader. Nonetheless, we are open to revision if the editor prefers a more neutral title such as: "Association between physical activity levels and peripheral muscle and diaphragm thickness: A cross-sectional study." We defer to the Editor's preference.

 2)Why is diaphragm chosen along with peripheral muscles? is it convenient to do US scan? or any valid reasons?

**Response**: The diaphragm is the primary inspiratory muscle and plays a critical role in respiratory and physical function. Physical activity, particularly aerobic exercise and breathing-intensive activities, has been proposed to influence diaphragmatic structure and function. However, observational evidence linking free-living PA to diaphragm thickness in healthy young adults is sparse. We included the diaphragm to extend the existing literature on the PA–muscle relationship beyond limb musculature and to test whether the diaphragm responds similarly. This rationale has been clarified in the Introduction.

 3)Its mentioned that change in the thickness of muscles changes as age

 progresses, but its not tested age wise. If the age group is restricted between 18-35 this

 question being raised is appropriate?

**Response**: The reviewer raises a fair point. Our study was intentionally restricted to 18–35-year-olds to minimise the confounding effect of age-related sarcopenia, which predominantly occurs after 50 years. Within this narrow age range, age-related muscle atrophy is unlikely to be a major driver of between-individual differences in muscle thickness, and our regression analysis (now added) confirms that age was not a significant predictor of muscle thickness in this sample. We have adjusted the manuscript to clarify that age-related change is a hypothesis for future longitudinal work, not a primary aim of this cross-sectional study.

 4)Method-Wide range of age with comorbidities included would give more insight or would have done on normal individuals. Provide clear insights with reasoner?

**Response**: We understand the reviewer's perspective. A broader age range or a sample with comorbidities would certainly provide additional clinical insights. However, for our primary research question — whether habitual PA is associated with muscle thickness in young healthy adults — restricting to 18–35 years without significant comorbidities was methodologically appropriate, as it reduces confounding by disease-related muscle wasting. Expanding to clinical populations with comorbidities would be valuable future work, and we have noted this explicitly in the Discussion as a direction for future research.

 5)In abstract and in title its mentioned cross-sectional study but in methodology its mentioned as single-centred randomized crossover trial. Is the study RCT? later changed? if changed permission from IRB would elucidate the readers?

**Response**: We sincerely apologise for this critical inconsistency. This was an error in the Methods section. The study was always designed and conducted as a cross-sectional observational study — single measurements at one time point, with no intervention or randomisation. The description "prospective single-centred randomised crossover trial" was an erroneous carryover from an earlier draft. The Ethics Committee approval (IEC2: 125/2022) and CTRI registration (CTRI/2022/10/046187) were obtained for an observational cross-sectional study. This has been corrected throughout the manuscript. We have also added a statement confirming this with the Ethics Committee.

 6)Reasoning for no change in the diaphragm muscle is not discussed. Discussion would have provided more insights.

**Response**: This is a valid and important point. We have expanded the Discussion to specifically address the null finding for the diaphragm. Possible explanations include: (1) the diaphragm is a tonic respiratory muscle that is active even during sedentary wakefulness, limiting the differential loading stimulus between PA groups; (2) the 13 MHz linear transducer used for limb muscles may have had suboptimal sensitivity for detecting small diaphragm changes without a curvilinear probe; (3) the S-IPAQ captures overall PA but does not specifically quantify breathing-intensive exercise (e.g., swimming or wind instrument playing) that might selectively load the diaphragm; and (4) the age-restricted sample (18–35 years) may have insufficient PA-related variance to detect diaphragm adaptation.

# Reviewer report: Reviewer response for version 2

**DOI:** 10.5256/f1000research.155807.r264818

**Published:** 2024-05-10

**Authors:** Dustin J Oranchuk

**Affiliations:** 1 Physical Medicine and Rehabilitation, University of Colorado Denver, Denver, Colorado, USA

General:

 I like the general direction of the study and the findings are somewhat interesting.

 The writing is understandable. However, the writing could be improved considerably. As an overall take, the writing is redundant and/or too wordy in places. While I do provide a few examples below, I encourage the authors to check the article throughout.

 The methods are missing many important details, the most pressing being the lack of a data analysis (how were the images analysed) and statistical analysis (what software was used, what tests were run and why etc.) This is extremely important and frankly shocking that they are currently missing…

 I really wanted to like the study, and it does have some strong points. However, the missing details and lacking analyses severely limit its use currently. While I do believe the authors can improve their work without any further data collection.

 Title:

 The title is good. The ‘A cross-sectional study’ is not needed, but the authors are free to leave this is they wish.

**Abstract:**

 ‘muscle build-up’ is an interesting way of stating it. However, I suggest changing it to ‘muscle mass’. If this change is made, please do so throughout the rest of the abstract and article.

 Indeed, two sentences later, the authors bring up ‘muscle thickness’ (a proxy for muscle mass).

 ‘In terms of occupation’ can be deleted as the following portion of the sentence is clear without the preface.

 ‘We acquired ultrasonography for…’. Perhaps the authors would be better served by simply writing ‘muscle thickness for muscles X, Y, Z were determined via ultrasonography’?

 Please use the ‘≤’ symbol, instead of “=<”

**Introduction:**

 The introduction is quite clear and mostly well written.

 In the second paragraph, the authors write ‘…like sarcopenia which is a major risk and early mortality’. Do the authors mean ‘…which is a major risk for early mortality.’

**Methods:**

 Good work including the exact type of study and the exact dates that the study was conducted over.

 Figure 1 is nice looking.

 Could the authors include reliability and/or validity statistics (and a citation) for the S-IPAQ? Obviously, questionnaires hold some error, but including these statistics could help to understand how limiting this approach is.

 While including sample size estimation is great, please include more details. Why as r1>0.4 selected? Do you have any similar studies that can be cited to support this number?

 Figure 2 is also nice.

 More detail is required for both the quadriceps and soleus measures. Specifically, where from distal to proximal were the scans obtained? For example, was the rectus femoris measured halfway between the anterior superior iliac spine and the base of the patella? Or some other landmarks? Was it 60% of the distance etc…? Same with the soleus, what landmarks and distances were used to find the desired region of each muscle.

 Same with the diaphragm? Personally I am much less familiar with how to scan the diaphragm. Please provide more details and perhaps a citation to support the method.

 What is the manufacturer information for the ultrasound machine?

 The authors also need a ‘data processing’ section where they describe how the images were analysed. For example, were the images analysed on the

 Maybe this is a peculiarity of the journal… but I do not see a ‘statistical analysis’ subsection.

 The authors MUST be clear regarding what software was used (figures looks like JASP), what tests were run (correlations, t-tests, ANOVAs etc.) and their details, and how the findings were/are interpreted (what constitutes a good correlation, what cutoff is considered statistical significance, how are confidence intervals interpreted etc.). Very important.

**Results:**

 The scatter plot(s) are nice and important to include, well done. It would be even better to include the 95% confidence intervals along with the Pearson’s correlation.

**Discussion:**

 I am a bit confused by the sentence that reads “The diaphragm thickness was 0.19 cm in LPA and 0.29 (p = 0.358) in PA.” That p-value is the same p-value given in table 2. Is that the correlation p-value? Or a t-test p-value between diaphragm thickness in LPA and PA? These are not the same and should not be used interchangeably.

 In reality, the authors can keep the correlational analysis, but should also perform an ANOVA (and post-hoc tests) to determine if the differences between physical activity tertiles (low, medium, and high) are significant. An effect size statistic (probably Cohen’s d) should also be used here.

 The above addition can help with the interpretation of the entire study.

 Conclusions should come after limitations.

Is the work clearly and accurately presented and does it cite the current literature?

No

If applicable, is the statistical analysis and its interpretation appropriate?

No

Are all the source data underlying the results available to ensure full reproducibility?

Yes

Is the study design appropriate and is the work technically sound?

Partly

Are the conclusions drawn adequately supported by the results?

Yes

Are sufficient details of methods and analysis provided to allow replication by others?

No

Reviewer Expertise:

Muscle morphology, physical performance, healthy ageing, strength and conditioning.

I confirm that I have read this submission and believe that I have an appropriate level of expertise to state that I do not consider it to be of an acceptable scientific standard, for reasons outlined above.

# Response

**Published:** 2026-03-07

**Authors:** suresh sukumar

**Affiliations:** Manipal College of Health Professions Manipal Academy of Higher Education, India

General:

 I like the general direction of the study and the findings are somewhat interesting.

 1)The writing is understandable. However, the writing could be improved considerably. As an overall take, the writing is redundant and/or too wordy in places. While I do provide a few examples below, I encourage the authors to check the article throughout.

**Response:** We thank the reviewer for this feedback. We have revised the manuscript throughout to reduce redundancy and improve conciseness. In particular, the Abstract, Introduction, and Discussion have been edited to eliminate repetitive phrasing.

 The methods are missing many important details, the most pressing being the lack of a data analysis (how were the images analysed) and statistical analysis (what software was used, what tests were run and why etc.) This is extremely important and frankly shocking that they are currently missing…

 I really wanted to like the study, and it does have some strong points. However, the missing details and lacking analyses severely limit its use currently. While I do believe the authors can improve their work without any further data collection.

**Response:** We thank the reviewer for this feedback. We have revised the manuscript throughout to reduce redundancy and improve conciseness. In particular, the Abstract, Introduction, and Discussion have been edited to eliminate repetitive phrasing.

 2)Title:

 The title is good. The ‘A cross-sectional study’ is not needed, but the authors are free to leave this is they wish.

**Response: **We appreciate the reviewer's flexibility. We have opted to retain the subtitle "A cross-sectional study" as it immediately communicates the study design to readers, which we believe adds clarity.

 3)Abstract:

 ‘muscle build-up’ is an interesting way of stating it. However, I suggest changing it to ‘muscle mass’. If this change is made, please do so throughout the rest of the abstract and article.

 Indeed, two sentences later, the authors bring up ‘muscle thickness’ (a proxy for muscle mass).

 ‘In terms of occupation’ can be deleted as the following portion of the sentence is clear without the preface.

 ‘We acquired ultrasonography for…’. Perhaps the authors would be better served by simply writing ‘muscle thickness for muscles X, Y, Z were determined via ultrasonography’?

**Response:** We accept all three suggestions and have implemented them throughout the abstract and manuscript.

 4)Please use the ‘≤’ symbol, instead of “=<”

**Response:** Corrected throughout the manuscript.

 5)Introduction:

 The introduction is quite clear and mostly well written.

 In the second paragraph, the authors write ‘…like sarcopenia which is a major risk and early mortality’. Do the authors mean ‘…which is a major risk for early mortality.’

**Response:** Yes, this was a typographic error. Corrected.

 6)Could the authors include reliability and/or validity statistics (and a citation) for the S-IPAQ? Obviously, questionnaires hold some error, but including these statistics could help to understand how limiting this approach is.

**Response: **We agree. The S-IPAQ has demonstrated test-retest reliability (ICC = 0.76) and acceptable criterion validity against accelerometry (Spearman's rho = 0.30–0.40) in international populations (Craig et al., Medicine & Science in Sports & Exercise, 2003). These statistics have been added to the Methods.

 7) While including sample size estimation is great, please include more details. Why as r1>0.4 selected? Do you have any similar studies that can be cited to support this number?

**Response: **A moderate correlation threshold of r > 0.4 was chosen based on published literature reporting PA–muscle thickness associations. Rostron et al. (2021) and Leblanc et al. (2015) reported correlations in the range of r = 0.35–0.55 between PA measures and muscle size. We selected r = 0.4 as a conservative estimate of the minimum clinically meaningful effect. This justification and citations have been added.

 8)More detail is required for both the quadriceps and soleus measures. Specifically, where from distal to proximal were the scans obtained? For example, was the rectus femoris measured halfway between the anterior superior iliac spine and the base of the patella? Or some other landmarks? Was it 60% of the distance etc…? Same with the soleus, what landmarks and distances were used to find the desired region of each muscle.

**Response:** We apologise for the insufficient detail. The following landmark descriptions have been added to the Methods.

 9)Same with the diaphragm? Personally I am much less familiar with how to scan the diaphragm. Please provide more details and perhaps a citation to support the method.

**Response: **We have expanded the diaphragm measurement section with additional methodological detail and have cited the standard reference (Boussuges et al., 2021; Goligher et al., 2015).

 10)What is the manufacturer information for the ultrasound machine?

**Response:** The ultrasound system used was a GE LOGIQ E9 (GE HealthCare, Chicago, IL, USA) with a 13 MHz linear transducer for peripheral muscles and a curvilinear transducer (3.5–5 MHz) for the diaphragm. This information has been added to the Methods.

 11)The authors also need a ‘data processing’ section where they describe how the images were analysed. For example, were the images analysed on the

 Maybe this is a peculiarity of the journal… but I do not see a ‘statistical analysis’ subsection.

**Response:** We agree this was a critical omission. A "Data Processing" subsection has been added to the Methods.

 12)The authors MUST be clear regarding what software was used (figures looks like JASP), what tests were run (correlations, t-tests, ANOVAs etc.) and their details, and how the findings were/are interpreted (what constitutes a good correlation, what cutoff is considered statistical significance, how are confidence intervals interpreted etc.). Very important.

**Response: **We agree this is a major omission. A dedicated Statistical Analysis subsection has been added.

 13)Results: The scatter plot(s) are nice and important to include, well done. It would be even better to include the 95% confidence intervals along with the Pearson’s correlation.

**Response:** Accepted. The scatter plots (Figure 3) have been updated to include 95% confidence interval bands around the regression lines.

 14)Discussion:

 I am a bit confused by the sentence that reads “The diaphragm thickness was 0.19 cm in LPA and 0.29 (p = 0.358) in PA.” That p-value is the same p-value given in table 2. Is that the correlation p-value? Or a t-test p-value between diaphragm thickness in LPA and PA? These are not the same and should not be used interchangeably.

**Response:** The reviewer is correct — the p = 0.358 was the correlation p-value from Table 2 (Pearson's r between PA and inspiratory diaphragm thickness), not a between-group t-test. Using it to describe group differences was inappropriate. We have corrected the Discussion to clearly attribute the p-value to the correlation analysis, and have now additionally provided the ANOVA results for between-group comparison of diaphragm thickness across PA tertiles.

 15)In reality, the authors can keep the correlational analysis, but should also perform an ANOVA (and post-hoc tests) to determine if the differences between physical activity tertiles (low, medium, and high) are significant. An effect size statistic (probably Cohen’s d) should also be used here.

 The above addition can help with the interpretation of the entire study.

**Response:** Accepted. One-way ANOVA with Tukey's post-hoc tests and Cohen's d effect sizes have been performed for all muscles across Low, Moderate, and High PA tertiles. These results are reported in the Results section and a new supplementary table has been added.

# Reviewer report: Reviewer response for version 2

**DOI:** 10.5256/f1000research.155807.r188228

**Published:** 2023-10-10

**Authors:** Suruliraj Karthikbabu

**Affiliations:** 1 KMCH College of Physiotherapy, Dr NGP Research and Educational Trust, Coimbatore, Tamil Nadu, India

Thank you for the opportunity to review the article titled “Active adults have thicker peripheral muscles and diaphragm: A cross-sectional study”.

 The authors found that adults with high physical activity had increased peripheral muscle thickness.

 I appreciate the author's efforts in conducting this study.  However, I find certain shortcomings in hypothesis testing and methodology, subsequently, interpreting the findings.

 The subjective physical activity level of patients during the past week was correlated with lower limb muscle thickness and diaphragm. I wonder why were the patients included in the study. The criterion seems they have the disease for which medical advice was sought. Please motivate me how their physical activity profile was reliable!!

 Short International Physical Activity Questionnaire (S-IPAQ) is a self-reported questionnaire that invites recall bias. Further, I’m afraid of how S-IPAQ is correlated with sensitivity tool ultrasonography. Ideally, the authors could have measured the physical activity using an objective measurement tool. Plus, justify using a parametric test, why not Spearman’s correlation co-efficiency!!

 Having a small sample size in a cross-sectional study limits subgrouping them based on physical activity level and further analysis. Please motivate me on how this work is a cross-over design when the readings are taken at a single time point!!  

 There is vast evidence supporting that exercise training and physical activity have a beneficial effect on muscle thickness. What is the rationale for generating a hypothesis when clinical trials already tested it?

 The hypothesis testing is not clear to me.

 The muscle strength can be substituted as muscle thickness. As age progresses, thickness reduces. The authors never tested this. To test this hypothesis, one needs a cohort study design.

 Age, smoking, and alcohol consumption being independent variables in this study never been analyzed and interpreted with stepwise multilinear regression analysis. I felt like the discussion section describes mostly the clinical variables, but lacks the reasoning for findings.             

 Many statements are not cited with appropriate references.

Is the work clearly and accurately presented and does it cite the current literature?

Partly

If applicable, is the statistical analysis and its interpretation appropriate?

Partly

Are all the source data underlying the results available to ensure full reproducibility?

Yes

Is the study design appropriate and is the work technically sound?

Partly

Are the conclusions drawn adequately supported by the results?

Partly

Are sufficient details of methods and analysis provided to allow replication by others?

Partly

Reviewer Expertise:

NA

I confirm that I have read this submission and believe that I have an appropriate level of expertise to confirm that it is of an acceptable scientific standard, however I have significant reservations, as outlined above.

# Response

**Published:** 2026-03-07

**Authors:** suresh sukumar

**Affiliations:** Manipal College of Health Professions Manipal Academy of Higher Education, India

Thank you for the opportunity to review the article titled “Active adults have thicker peripheral muscles and diaphragm: A cross-sectional study”.

 The authors found that adults with high physical activity had increased peripheral muscle thickness.

 I appreciate the author's efforts in conducting this study.  However, I find certain shortcomings in hypothesis testing and methodology, subsequently, interpreting the findings.

 1)The subjective physical activity level of patients during the past week was correlated with lower limb muscle thickness and diaphragm. I wonder why were the patients included in the study. The criterion seems they have the disease for which medical advice was sought. Please motivate me how their physical activity profile was reliable!!

**Response:** We appreciate this concern. The participants were recruited from individuals waiting for routine radiological screening, not from patients seeking treatment for a specific disease. Importantly, all participants were screened and excluded if they had conditions that could directly affect muscle thickness (e.g., recent trauma, orthopaedic interventions, chronic cardio-pulmonary disease, bed-ridden or paralysed status, osteoarthritis). The remaining participants were otherwise healthy young adults (18–35 years). This opportunistic sampling from a radiology waiting area is an established and ethically efficient method in observational studies, as it provides access to a diverse population while minimising additional burden.

 Regarding reliability of physical activity profiles: the S-IPAQ was administered at the same visit as the ultrasound scan, ensuring the self-report was concurrent with the physical measurement. Studies have validated S-IPAQ against accelerometry in similar age groups, confirming acceptable criterion validity.

 2)Short International Physical Activity Questionnaire (S-IPAQ) is a self-reported questionnaire that invites recall bias. Further, I’m afraid of how S-IPAQ is correlated with sensitivity tool ultrasonography. Ideally, the authors could have measured the physical activity using an objective measurement tool. Plus, justify using a parametric test, why not Spearman’s correlation co-efficiency!!

**Response:** We acknowledge that the S-IPAQ carries inherent recall bias as all self-report instruments do. Objective tools such as accelerometry or pedometry would have been preferable; however, they were not feasible within the resource and time constraints of this study. Despite this limitation, S-IPAQ is the most widely used and internationally validated brief PA questionnaire and has demonstrated moderate to good concurrent validity against objective measures across multiple studies (Craig et al., 2003; Ekelund et al., 2006). We have now explicitly acknowledged this limitation in the Discussion section.

 Regarding the choice of Pearson's correlation: the assumption of normality was tested prior to analysis. The muscle thickness values were approximately normally distributed within the sample, justifying the use of a parametric test. Nonetheless, in **response **to this comment, we have now additionally reported Spearman's rho alongside Pearson's r for all correlations in Table 2 to provide a non-parametric alternative.

 3)Having a small sample size in a cross-sectional study limits subgrouping them based on physical activity level and further analysis. Please motivate me on how this work is a cross-over design when the readings are taken at a single time point!! 

**Response:** We fully accept the reviewer's correction regarding study design. The original labelling of the study as a "prospective single-centred randomised crossover trial" was an error — this was a cross-sectional observational study with measurements taken at a single time point. We sincerely apologise for this inconsistency and have corrected the study design description throughout the manuscript (Abstract, Methods, and wherever referenced). The Ethics Committee approval was obtained for an observational cross-sectional study; we have clarified this in the text.

 Regarding sample size: 91 participants is adequate for correlation analysis. Our sample size was calculated to detect a moderate correlation (r > 0.4) at 95% confidence with 80% power, yielding a requirement of 84 participants (Bujang & Baharum, 2016). Our sample of 91 exceeds this. We acknowledge that subgroup analysis is limited, and have noted this as a limitation.

 4)There is vast evidence supporting that exercise training and physical activity have a beneficial effect on muscle thickness. What is the rationale for generating a hypothesis when clinical trials already tested it?

**Response:** While the general benefit of exercise on muscle hypertrophy is well established from clinical trials, the specific relationship between habitual free-living PA levels (not supervised exercise interventions) and ultrasound-measured peripheral muscle thickness — including the diaphragm — in a young adult South Asian population using S-IPAQ classification has not been well characterised. Most existing RCTs investigate the effect of structured exercise programs, which differ meaningfully from self-reported habitual PA. Additionally, observational evidence linking the diaphragm thickness to PA level is sparse. Our study addresses this observational gap in a distinct population, providing normative data that can inform clinical and public health practice.

 5)The hypothesis testing is not clear to me.

**Response:** We apologise for the lack of clarity. We have rewritten the hypothesis section at the end of the Introduction to explicitly state: (1) the primary hypothesis — higher levels of self-reported physical activity will be positively associated with greater ultrasound-measured peripheral muscle thickness (quadriceps and soleus); and (2) the secondary hypothesis — the diaphragm thickness will also show a positive association with physical activity level.

 6)The muscle strength can be substituted as muscle thickness. As age progresses, thickness reduces. The authors never tested this. To test this hypothesis, one needs a cohort study design.

**Response:** We agree that the relationship between age and muscle thickness deserves a dedicated longitudinal cohort study design. This is beyond the scope of the current cross-sectional work. Our study was intentionally restricted to adults aged 18–35 years to minimise age-related confounding (sarcopenic muscle loss being more prominent above 40–50 years). Within this narrow age range, age-related atrophy is minimal, making cross-sectional analysis appropriate for the primary aim of correlating PA levels with muscle thickness. We have acknowledged the need for cohort studies to investigate age-related changes in the Discussion and Limitations sections. Regarding muscle strength as a proxy: strength testing was not performed in this study; however, muscle thickness measured by ultrasound is a valid and accepted surrogate for muscle mass and has been shown to correlate with strength in prior research.

 7)Age, smoking, and alcohol consumption being independent variables in this study never been analyzed and interpreted with stepwise multilinear regression analysis. I felt like the discussion section describes mostly the clinical variables, but lacks the reasoning for findings.       

**Response:** This is an important point. We have now conducted a stepwise multiple linear regression analysis with muscle thickness as the dependent variable and PA level, age, smoking status, and alcohol consumption as independent variables. The results confirm that PA level remains a significant predictor of muscle thickness after adjusting for these confounders. These results have been added to the Results section and interpreted in the Discussion.    

 8)Many statements are not cited with appropriate references.

**Response:** We have conducted a thorough review of the manuscript and added appropriate citations where they were missing. All factual statements in the Introduction and Discussion are now supported by references.

# Reviewer report: Reviewer response for version 1

**DOI:** 10.5256/f1000research.148494.r194233

**Published:** 2023-08-30

**Authors:** Małgorzata Pałac

**Affiliations:** 1 Musculoskeletal Elastography and Ultrasonography Laboratory, Institute of Physiotherapy and Health Sciences, The Jerzy Kukuczka Academy of Physical Education, Katowice, Poland

I appreciate the work on this interesting and inspiring study. The article analyzes the muscles thickness of the lower limb and diaphragm with the level of physical activity. The study included a considerable number of participants. However, the article needs improvement.

 1. Inaccurate information in methodology.

- Not clear breathing maneuver during the diaphragm measurement:

*“Using M-mode, the diaphragmatic thickness was determined. Tdi, ee (Diaphragmatic thickness at end-expiration) and Tdi, pi (peak inspiration) measurements were already taken on consecutive breaths, which were seen in a single M-mode image. The diaphragmatic thickness was measured before normal inhalation and after complete exhalation.” (methods)*

 In the first sentence diaphragm thickness was measured at the end-expiration and in peak inspiration. In the second sentence diaphragm thickness was measured before normal inhalation and after complete exhalation. It is not clear which information is true.

- Secondly, no information was provided about where exactly the thickness measurements were taken from the images of muscles. This is not clear in Fig. 2.
- Was the only one value collected from each photo? It would be better to describe it in the methodology section.
- *“The chest wall was aligned perpendicularly with a 13-MHz linear array transducer.”*   Is the diaphragm definitely measured by a linear probe?
- Moreover, it would be appropriate to add information about the subjects' BMI.
- The results include information about smoking and drinking alcohol. The method lacks information on how the data was collected.
- Incorporating the interpretation of correlation results into the methodology and subsequently discussing them in the results and discussion sections is valuable. This approach contributes to a more accurate and insightful analysis of the results.
- *“To measure the quadriceps”* section is described in the subchapter. " *To measure the soleus and diaphragm*" is listed as sub-items in the subchapter to “ *measure the quadriceps section*”. It is better to divide all descriptions to subchapters. The information from “ *muscle thickness”* subsection in the methodology seem to be redundant.

It is important to provide sufficient details of methods to allow replication and analysis by others.

 2. The introduction and discussion contains interesting information, but it is good to confirm it with a citation. The information then looks more reliable, e.g.

- *“Muscle mass and strength are predictors of performance enhancement and ability to work in adults and mobility functions in the elderly population.” *(introduction)
- *“Furthermore, peripheral muscle mass and strength are associated with chronic diseases like sarcopenia which is a major risk and early mortality”. *(introduction)
- *“The reason for selecting the quadriceps, soleus in the lower limb, and the diaphragm for the study were that many researchers have found that there is a change in muscle thickness as age progresses, and it differs with sex as well.” *(discussion)         The sentence seems intended to explain why these particular muscles were selected for analysis. If that was the aim, what is missing is the citation indicating that these particular muscles change in thickness with age.

3. In introduction there is sentence: “ *Experimental studies have administered several interventions to address the increasing burden of physical inactivity and SB.”*

 Unfortunately, this information was not given in article.

 4. The hypotheses are interesting, but they do not fully align with the research in this article.

 5. " *Anecdotal evidence*" that are not scientifically proven should not be described, e.g.:

*“Though anecdotal evidence claims a bidirectional relationship between physical inactivity and peripheral muscle strength or thickness, observational studies establishing the relationship are lacking.”* (introduction)

 If research has been done on this subject but not published, it is worth mentioning it. If not, it may be better to add this kind of suggestion in the discussion (if needed).

 6. It is a very excellent idea to add a flow diagram regarding the qualification stage of participants. Unfortunately, the flow diagram needs improvement. It might look more aesthetically pleasing if the shape and size of the elements were unified.

 In the flow diagram, the initial number of participants was 91, which is the same as the final number. Is it the same number for sure? Below "patients screened", "bedridden patients not screened for the study" is mentioned. This suggests that some people were excluded for this reason. However, the number of people excluded from the study for this reason was not reported. I think that in the flow chart it is worth specifying the initial number of people at the top. Then, the number of those excluded due to the exclusion criterion (as listed in the methodology: recent trauma, orthopedic interventions, bedridden, paralyzed, osteoarthritis, and other chronic diseases of the heart and lung).

 7. The instruments need to be better characterized, with the manufacturer, city, state (if applicable), and country of manufacture, including the programs for data analysis used.

 8. Some photos could have a better quality (Fig. 2) and not be cropped. I suggest transferring images to a computer, then choosing a clearly visible image. It would look better.

 9. English in some places need improvement, e.g.

- *“The study included 91 patients aged 18 to 35 years with N = 78 male subjects with mean age and standard deviation of 27.954 years 4.67 and N = 13 females with mean age and standard deviation of 27.978 years 4.67.”* (results)
- *“According to previoys studies, men’s skeletal muscle degradation is correlated with age at about 27 years of age. With this clause, we have restricted our study age group to between 18-35 years.”* (discussion)

In these sentences authors should choose “ *age*” or “ *years*”.

- Additionally, if a standard deviation is given for the age, it is sufficient for the average number of ages to be rounded to the nearest whole number (maximum of one decimal place). It looks more readable.
- Moreover, in some sentences it says *the "diaphragm thickness"*, in others the " *diaphragm muscle thickness"* (discussion). The diaphragm thickness is sufficient.

Considering this I recommend to send the manuscript to a fluent English-speaking person and ask them to correct the linguistic aspect of the manuscript.

 10. Some information is redundant or repeated:

- The number and age of participants are repeated twice in the results section (3 lines apart).

To simplify and not repeat the information, the first sentences from the results section can be placed in the *baseline characteristic* section.

- Secondly, I don't see the purpose of providing employment information. Maybe it would be a good idea if the results were divided into two groups to show different correlations between employed and unemployed people. In the context of this work, perhaps a better question would be: Does the daily work performed by the participants involve physical or office work?
- Thirdly, results given in the table are repeated in the text. This is not advisable. Moreover, several results from the text do not coincide with the results from the tables.

11. Table 1 needs improvement.

- I would suggest changing the title of the table, e.g. Patients characteristics.
- Also, remove the occupation information.
- Some values are given to a whole number, some to 1 decimal place, some to 2 decimal places, others with 3 decimal places. This should be standardized.
- There is a lack of explanation of abbreviations in the tables. Some of them are explained in the text it would be clearer to the reader if they were also placed below the table (the same as MET was explained).

12. Some of the sentences in the article are incorrect or stylistically/grammatically wrong which sometimes makes them seem unclear e.g.

- * “Out of 91 patients, 30 were found to regularly smoke, and six had drinking habits after questions regarding lifestyle characteristics including smoking and drinking were asked*.” (results)
- *“This increase in diaphragm thickness may result in increased inspiratory muscle efficiency or improved pulmonary mechanics, or both.” *(discussion)
- *“Recent studies have shown and proved that diaphragm muscle thickness changes with increased PA, e.g. weight training.” *(discussion)

Just use one shown or proved.

- *“We required 91 samples to achieve a moderate correlation (r1 > 0.4) at an alpha level of 95% and an 80% strength.”*

Is it certain that the version " *rl*" is the correct one?

- *“With all these factors as constants and variables, our study shows that with an increase in PA, there is a significant increase in the quadriceps (…).” *(discussion)

The sentence is misleading. Muscle thickness was not studied with increasing physical activity. It was written in a better form in the conclusions: “ *Peripheral muscle thickness has been found to positively correlate with physical activity levels.”*

Is the work clearly and accurately presented and does it cite the current literature?

Partly

If applicable, is the statistical analysis and its interpretation appropriate?

Partly

Are all the source data underlying the results available to ensure full reproducibility?

Partly

Is the study design appropriate and is the work technically sound?

Partly

Are the conclusions drawn adequately supported by the results?

Partly

Are sufficient details of methods and analysis provided to allow replication by others?

Partly

Reviewer Expertise:

Respiratory muscle ultrasonography, Physiotherapy, Shear wave elastography

I confirm that I have read this submission and believe that I have an appropriate level of expertise to state that I do not consider it to be of an acceptable scientific standard, for reasons outlined above.

# Response

**Published:** 2023-09-08

**Authors:** suresh sukumar

**Affiliations:** Manipal College of Health Professions Manipal Academy of Higher Education, India

We are grateful for the comments and suggestions provided. we have tried to incorporate the changes and justified the same. 

1.  Inaccurate information in methodology.

- Not clear breathing manoeuvre during the diaphragm measurement: In the first sentence diaphragm thickness was measured at the end-expiration and in peak inspiration. In the second sentence diaphragm thickness was measured before normal inhalation and after complete exhalation. It is not clear which information is true.

the diaphragmatic thickness was determined as the distance between the diaphragmatic pleura and the peritoneum at end-expiration (Tdi,ee) and peak inspiration (Tdi,pi). To be more specific we have mentioned as before normal inspiration.

- Was the only one value collected from each photo? It would be better to describe it in the methodology section.

Yes only one value was measured – inspiration and expiration values.

- *“The chest wall was aligned perpendicularly with a 13-MHz linear array transducer.”*   Is the diaphragm definitely measured by a linear probe?

Yes a high frequency linear probe is used for better resolution and a wide field of view.

- Moreover, it would be appropriate to add information about the subjects' BMI.

      Unfortunately we haven’t collected the body mass index data .

- The results include information about smoking and drinking alcohol. The method lacks information on how the data was collected.

The data was collected while the patient had to fill out the questionnaire.

- *To measure the quadriceps”* section is described in the subchapter. " *To measure the soleus and diaphragm*" is listed as sub-items in the subchapter to “ *measure the quadriceps section*”. It is better to divide all descriptions to subchapters. The information from “ *muscle thickness”* subsection in the methodology seem to be redundant.

The changes shall be done as instructed.

  

-  The introduction and discussion contains interesting information, but it is good to confirm it with a citation. The information then looks more reliable,

Definitely we’ll work on that.

- . " *Anecdotal evidence*" that are not scientifically proven should not be described, e.g.:
*“Though anecdotal evidence claims a bidirectional relationship between physical inactivity and peripheral muscle strength or thickness, observational studies establishing the relationship are lacking.”* (introduction)
  If research has been done on this subject but not published, it is worth mentioning it. If not, it may be better to add this kind of suggestion in the discussion (if needed).

Sure will do the changes.

  

- In the flow diagram, the initial number of participants was 91, which is the same as the final number. Is it the same number for sure? Below "patients screened", "bedridden patients not screened for the study" is mentioned. This suggests that some people were excluded for this reason. However, the number of people excluded from the study for this reason was not reported. I think that in the flow chart it is worth specifying the initial number of people at the top. Then, the number of those excluded due to the exclusion criterion (as listed in the methodology: recent trauma, orthopedic interventions, bedridden, paralyzed, osteoarthritis, and other chronic diseases of the heart and lung).

Yes we screened 91 excluding the bed ridden patients and the procedure was focused on these patients. But I shall add the initial number in the beginning .

  

- English in some places need improvement, e.g.

- *“The study included 91 patients aged 18 to 35 years with N = 78 male subjects with mean age and standard deviation of 27.954 years 4.67 and N = 13 females with mean age and standard deviation of 27.978 years 4.67.”* (results)
- *“According to previoys studies, men’s skeletal muscle degradation is correlated with age at about 27 years of age. With this clause, we have restricted our study age group to between 18-35 years.”* (discussion)

The study comprised 91 individuals between the ages of 18 and 35. There were 78 male subjects (mean age, standard deviation, 27.954 years) and 13 female subjects (mean age, standard deviation, 27.978 years).

 Previous research has shown that men's skeletal muscle deterioration starts to increase at the age of 27. This provision limits the study age range to those between the ages of 18 and 35.

- Secondly, I don't see the purpose of providing employment information. Maybe it would be a good idea if the results were divided into two groups to show different correlations between employed and unemployed people. In the context of this work, perhaps a better question would be: Does the daily work performed by the participants involve physical or office work?

The occupation data was only collected to have a brief understanding about patient’s PA and sedentary lifestyle.

  

- Some of the sentences in the article are incorrect or stylistically/grammatically wrong which sometimes makes them seem unclear e.g.

- * “Out of 91 patients, 30 were found to regularly smoke, and six had drinking habits after questions regarding lifestyle characteristics including smoking and drinking were asked*.” (results)
- *“This increase in diaphragm thickness may result in increased inspiratory muscle efficiency or improved pulmonary mechanics, or both.” *(discussion)
- *“Recent studies have shown and proved that diaphragm muscle thickness changes with increased PA, e.g. weight training.” *(discussion)
- Corrections :
- In responses to inquiries on lifestyle factors including drinking and smoking, it was discovered that 30 of the 91 patients smoked frequently and 6 had drinking habits. (results)
- The rise in diaphragm thickness might lead to improved pulmonary mechanics, enhanced inspiratory muscle efficiency, or even both. (discussion)
-  "Recent research has shown that diaphragm thickness changes with increased PA, such as weight training." (discussion)

# Reviewer report: Reviewer response for version 1

**DOI:** 10.5256/f1000research.148494.r188229

**Published:** 2023-08-25

**Authors:** Ranganath Gangavelli

**Affiliations:** 1 GITAM School of Physiotherapy, Gandhi Institute of Technology and Management (GITAM), Visakhapatnam, Andhra Pradesh, India

I appreciate the author's efforts in conducting this study. It is interesting to study the impact of physical activity and its impact on muscle thickness. The Introduction is well structured. However, there are a few aspects that need serious consideration.

1. The study is reported as a cross-sectional design in the Abstract. However, in the Methods section of the Manuscript, it is mentioned as a randomized crossover design approved by the  Institutional Ethics Committee. Further, the trial was registered as an interventional study in CTRI. Please justify the discrepancy. However, no medical/ therapeutic intervention was reported in the manuscript except for diagnostic investigation.
2. The hypothesis statement is not clear. The muscle Strength measures are never reported in the manuscript. Are the thickness measures equated to strength? The word "change" is confusing. Are the authors measuring the change in this study? Which of the three designs mentioned earlier is adopted in the study and is used to test the hypothesis? Or is it a cohort study to determine the "change" in the thickness of muscles as age progresses in an individual, as stated in the 2nd hypothesis? However, only a single timepoint for thickness measure was reported in the manuscript.
3. Regarding participants: Please justify the reason for recruiting the Patients while the data could be collected from asymptomatic volunteers with self-reported physical activity levels. Could the underlying health condition (not stated in the manuscript) influence the data? Details/reports of provisional or final diagnoses will help interpret the data.
4. The procedure is expressed in the future tense.
5. The ankle's position while measuring the soleus's thickness is repetitive.
6. Please justify obtaining the data on smoking and alcohol use. It is not discussed.
7. The expression of the result is inconsistent. 
8. The reported diaphragm thickness is quite less than earlier reports ^1^. Please discuss the reasons for the difference in recording. The population seems to be the same. 
9. Grammar and spelling checks are required in the Results and Discussion sections. 
10. Body Mass Index, along with Physical activity level, may have an impact on the thickness measures. Please report if the data is available.

Is the work clearly and accurately presented and does it cite the current literature?

Partly

If applicable, is the statistical analysis and its interpretation appropriate?

Partly

Are all the source data underlying the results available to ensure full reproducibility?

Partly

Is the study design appropriate and is the work technically sound?

Partly

Are the conclusions drawn adequately supported by the results?

Partly

Are sufficient details of methods and analysis provided to allow replication by others?

Yes

Reviewer Expertise:

Musculoskeletal Health, Mobilization, Pain.

I confirm that I have read this submission and believe that I have an appropriate level of expertise to state that I do not consider it to be of an acceptable scientific standard, for reasons outlined above.

## References

[1] : Reproducibility of diaphragm thickness measurements by ultrasonography in patients on mechanical ventilation. *World J Crit Care Med* .2017;6(4) : 10.5492/wjccm.v6.i4.185 185-189 10.5492/wjccm.v6.i4.185 29152465 PMC5680345

# Response

**Published:** 2023-09-08

**Authors:** suresh sukumar

**Affiliations:** Manipal College of Health Professions Manipal Academy of Higher Education, India

We are grateful for the comments and suggestions.We have tried to incorporate the changes and have justified for the same.  

1. The study is reported as a cross-sectional design in the Abstract. However, in the Methods section of the Manuscript, it is mentioned as a randomized crossover design approved by the  Institutional Ethics Committee. Further, the trial was registered as an interventional study in CTRI. Please justify the discrepancy. However, no medical/ therapeutic intervention was reported in the manuscript except for diagnostic investigation.

The study is an observational study to see the changes within the muscles with respect to physical activity.  Since the study includes various procedures and activity we have mentioned it as interventional study. Furthermore the RCT design changes shall be made.

1. The hypothesis statement is not clear. The muscle Strength measures are never reported in the manuscript. Are the thickness measures equated to strength? The word "change" is confusing. Are the authors measuring the change in this study? Which of the three designs mentioned earlier is adopted in the study and is used to test the hypothesis? Or is it a cohort study to determine the "change" in the thickness of muscles as age progresses in an individual, as stated in the 2nd hypothesis? However, only a single timepoint for thickness measure was reported in the manuscript.

The muscle strength measurements are not mentioned here but instead we have measured the thickness. Also Muscle strength depends on muscle quality as well as muscle quantity (e.g. muscle volume including muscle thickness). 

 To simplify the word change here we have measured the muscle thickness of a physically active individual and sedentary lifestyle individual to see the changes in the muscle thickness with respect to physical activity.

 It’s a combination of cross sectional and interventional study since we have taken a period of 7 days for the measurement of muscle which would be considered a short span of time to be determined as cohort study.

1. Regarding participants: Please justify the reason for recruiting the Patients while the data could be collected from asymptomatic volunteers with self-reported physical activity levels. Could the underlying health condition (not stated in the manuscript) influence the data? Details/reports of provisional or final diagnoses will help interpret the data.

We have chosen the patients from the KMC hospital as it was more feasible to collect data from them. Also after screening the patient we have excluded patients with recent trauma, orthopedic interventions, bedridden, paralyzed, osteoarthritis, and other chronic diseases of the heart and lungs which can hamper the muscle thickness.

1. The procedure is expressed in the future tense.

Apologies for the inconvenience. Corrections shall be done.

  

1. The ankle's position while measuring the soleus's thickness is repetitive

Corrections shall be done.

1. Please justify obtaining the data on smoking and alcohol use. It is not discussed.

The motive of obtaining the smoking and alcohol consumption details was basically to see if it hampers the change in the muscle thickness of an individual.

1. The expression of the result is inconsistent. 

We shall work on that and improvise it.

1. The reported diaphragm thickness is quite less than earlier reports ^1^ . Please discuss the reasons for the difference in recording. The population seems to be the same

The measurement may vary with patients, the inspiration and expiration rate may change too since it’s a self- reported study minor differences can occur.

1. Grammar and spelling checks are required in the Results and Discussion sections. 

Definitely changes will be made.

1. Body Mass Index, along with Physical activity level, may have an impact on the thickness measures. Please report if the data is available.

 Unfortunately we haven’t taken the body mass index data with PA.
